# *Crocodylus porosus*: a potential source of anticancer molecules

**DOI:** 10.1136/bmjos-2019-100040

**Published:** 2020-10-27

**Authors:** Shareni Jeyamogan, Naveed Ahmed Khan, K Sagathevan, Ruqaiyyah Siddiqui

**Affiliations:** 1 Department of Biological Sciences, Sunway University, Bandar Sunway, Selangor, Malaysia; 2 Department of Biology, Chemistry and Environmental Sciences, American University of Sharjah, University City, Sharjah, United Arab Emirates; 3 Science and Technology, Sunway College, Bandar Sunway, Selangor, Malaysia

**Keywords:** cancer, anticancer molecules, cytotoxicity, growth inhibition

## Abstract

**Background:**

Cancer remains a global threat resulting in significant morbidity and mortality despite advances in therapeutic interventions, suggesting urgency for identification of anticancer agents. Crocodiles thrive in polluted habitat, feed on germ-infested meat, are exposed to carcinogenic heavy metals, are the very few species to survive the catastrophic Cretaceous–Paleogene extinction event, yet have a prolonged lifespan and rarely been reported to develop cancer. Therefore, we hypothesised that animals living in polluted environments such as crocodiles possess anticancer molecules/mechanisms.

**Methods:**

*Crocodylus porosus* was procured, blood collected, dissected and lysates prepared from internal organs. Organ lysates and sera were tested for growth inhibition, cytotoxic effects and cell survival against HeLa, PC3 and MCF7 cells and subjected to liquid chromatography mass spectrometry. RNA transcriptome analysis and differential gene analysis were performed using Galaxy Bioinformatics.

**Results:**

Sera exhibited potent growth inhibition and cytotoxic effects against cancer cells. 80 molecules were detected from *C. porosus* and 19 molecules were putatively identified. Additionally, more than 100 potential anticancer peptides were identified from sera using bioinformatics based on peptide amino acid composition, binary profile, dipeptide composition and pseudo-amino acid composition. Following transcriptome analysis, 14 genes in treated HeLa cells, 51 genes in treated MCF7 cells and 2 genes in treated PC3 cells, were found to be expressed, compared with untreated controls.

**Conclusion:**

Animals residing in polluted milieus are an unexploited source for prospective pharmaceutical drugs, and could lead to identification of novel antitumour compound(s) and/or further understanding of the mechanisms of cancer resistance.

**Strengths and limitations of this study**

- To our knowledge, this is the first detailed study performed to investigate the presence of anticancer activity, anticancer molecules and peptides from the serum of *Crocodylus porosus* as well as the differential gene expression of cancer cells treated with crocodile serum.
- Adequate steps were taken to limit the risk of bias: the primary end point was prespecified, and the number of samples to be tested was determined beforehand to ensure sufficient clarity.
- Experiments were substantiated by repetition, under a range of conditions that demonstrate the robustness of the effects observed. The data are representative as the mean±SE of three independent experiments.
- The present study involved serum collection from one crocodile and data obtained from subsequent experiments conducted, are hypothesis-confirming experiments and should be interpreted as such, being in the exploratory phase of research. Further validation of results is needed.

## Introduction

An significant increase in the number of cancer cases and cancer deaths was observed from 2000 to 2018 despite advances in therapeutic interventions and supportive care.^1–3^ GLOBOCAN reports demonstrated an increase in the number of cancer cases from 10.1 million in 2000 to 18.1 million in 2018 while the number of deaths has increased from 6.2 million in 2000 to 9.6 million cases in 2018.^1–3^ Therefore, there is an urgent need for the discovery and development of new and efficient anticancer agents.

Animals such as crocodiles inhabit unsanitary and polluted environments, feed on rotten meat which is present with numerous pathogenic microbes, are continuously exposed to heavy metals that are genotoxic and carcinogenic such as arsenic, nickel, zinc, cadmium, cobalt, mercury, selenium, lead and chromium^4–7^ and are also the very few species to survive the Cretaceous–Paleogene mass extinction despite being exposed to extreme levels of radiation.^4 8–10^ Despite all of the above, these animals have prolonged lifespan and rarely develop cancer.

Our lab has recently hypothesised that animals living in polluted environments such as crocodiles possess mechanisms or molecules against cancer development. In support, our previous studies showed that organ lysates of *Crocodylus palustris* inhibited the growth and demonstrated killing effects against PC3 cells.^4^ Moreover, previous studies have shown that bile products of crocodile (*Crocodylus siamensis*) inhibited the growth of cancer cells such as human cholangiocarcinoma cells that include Mz-ChA-1 cells, QBC939 cells, Sk-ChA-1 cells,^11 12^ A2780 human ovarian cancer cells^13^ and human gastric adenocarcinoma BGC823 cells.^14^Furthermore, it was shown that leucrocin I, peptide isolated from the blood extracts of *C. siamensis,* was also capable of inducing cell death.^15^ Here we have undertaken a detailed study to investigate the presence of anticancer activity, potential anticancer molecules and peptides from the serum of *Crocodylus porosus*. Furthermore, we also investigated the differential gene expression of cancer cells treated with crocodile serum. The discovery of anticancer molecule/mechanism from crocodile can pave the way for the discovery and development of therapeutic interventions.

## Materials and methods

### Ethics Committee consent and procurement of crocodile

**Figure 1:**
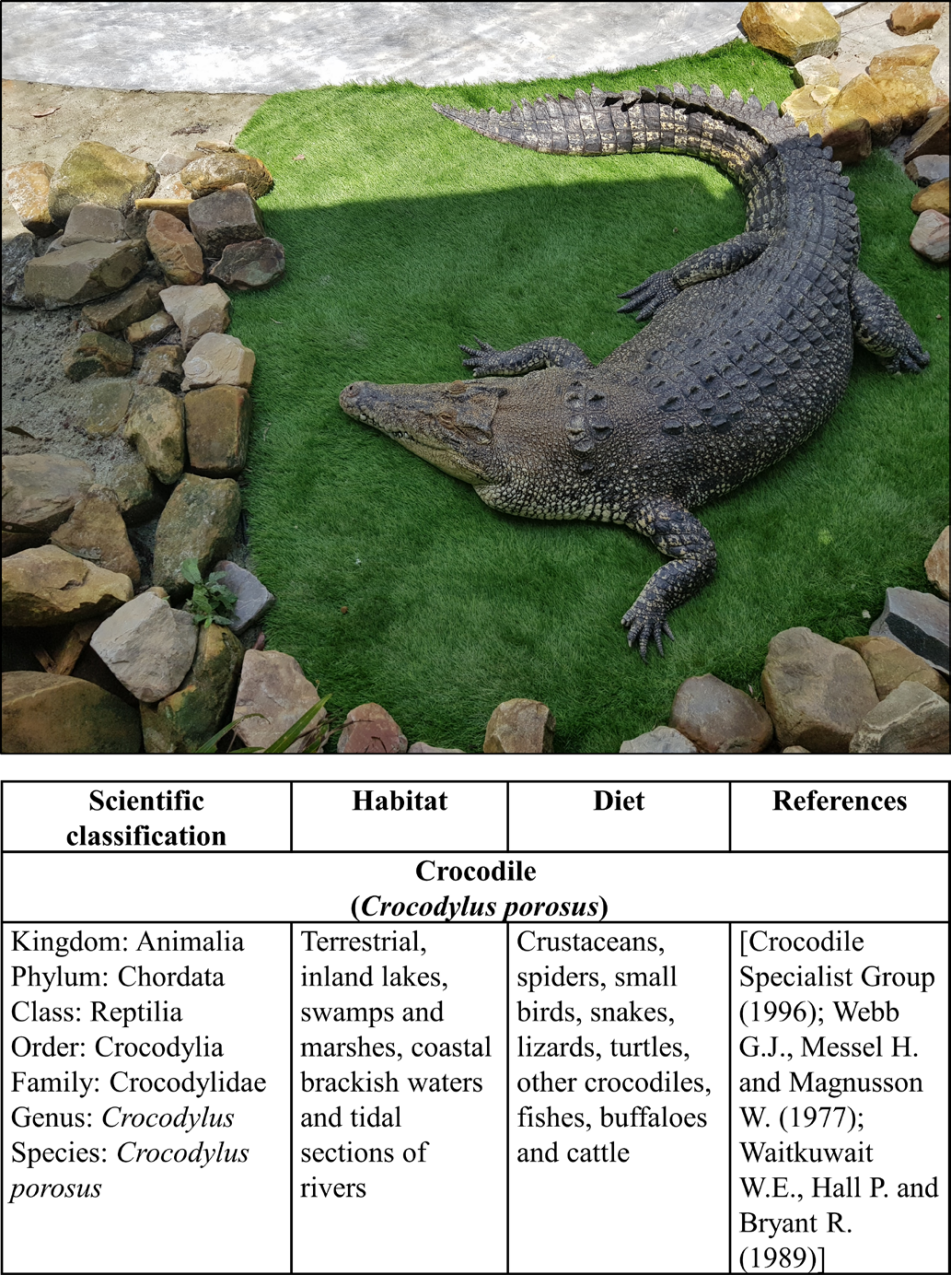
The species, scientific classification, habitat and diet of crocodile (*Crocodylus porosus*).

The saltwater crocodile, *C. porosus* was donated by a Convention on International Trade in Endangered Species of Wild Fauna and Flora (CITES)-registered crocodile farm (figure 1). Handling of the animal, anaesthesia and dissection of the internal organs were carried out by the pathologist at the farm. Personnel at the crocodile farm routinely perform such procedures.

### Chemicals and reagents

The chemicals and reagents used for the assays in this manuscript comprise of lactate dehydrogenase (LDH) cytotoxicity kit (Roche Diagnostics, Indianapolis, USA), Trypan blue (Merck Millipore, Germany), pronase enzyme from *Streptomyces griseus* (Cat. No. 10165921001; Roche Applied Science, Switzerland), Quick Start Bradford dye and Quick Start bovine serum albumin standards (Bio-Rad Laboratories, Hercules, California, USA), protease inhibitor (Problock Gold Mammalian, St Louis, Missouri, USA), Roswell Park Memorial Institute 1640, L-glutamine solution, fetal bovine serum (FBS) and trypsin 2.5% solution (Serana, Pessin, Germany), penicillin streptomycin antibiotic solution (Life Technologies, Carlsbad, California, USA), minimum essential medium non-essential amino acid (MEM NEAA) solution (Sigma Aldrich, St Louis, Missouri, USA, high-performance liquid chromatography (HPLC)-graded methanol, HPLC-graded formic acid and HPLC-graded acetonitrile (Merck Group, Darmstadt, Germany), ultra-pure deionised MiLi-Q water (EMD Millipore, Burlington, USA), ammonium bicarbonate, trifluoroethanol, dithiothreitol and iodoacetamide (Nacalai Tesque, Kyoto, Japan) unless stated otherwise.

### Sample collection

**Figure 2:**
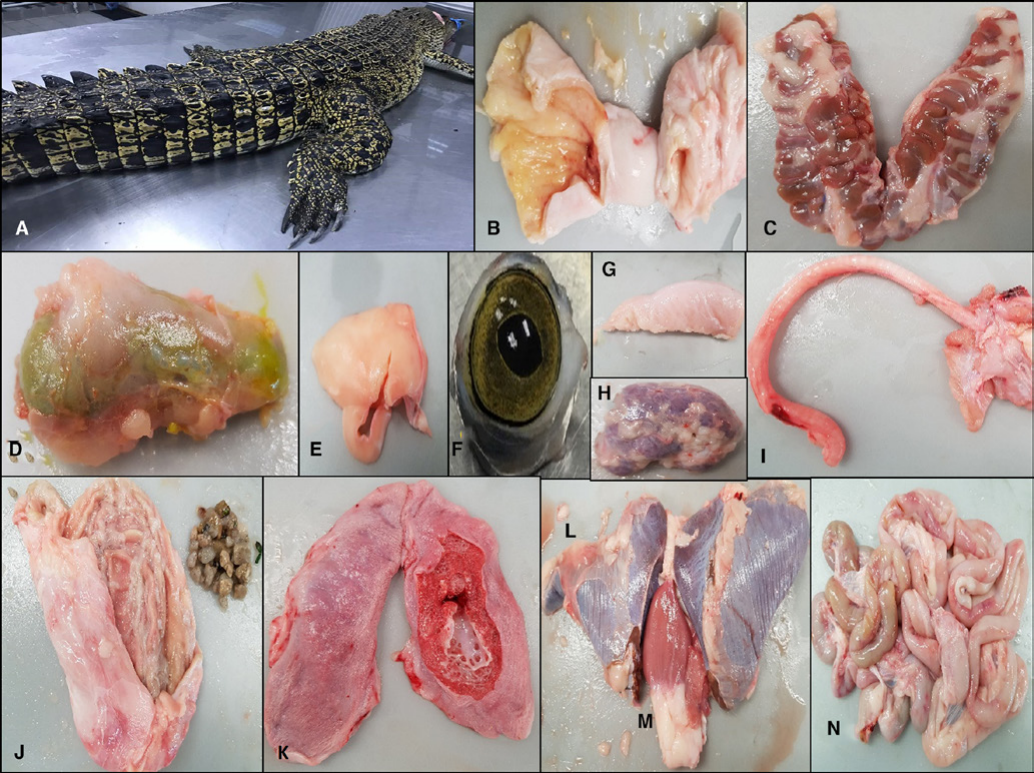
Dissection of crocodile (*Crocodylus porosus*). A female crocodile, of species *C. porosus* was dissected (A). The various body organs of the crocodile were dissected out, including (B) large intestines and caecum, (C) kidneys, (D) gall bladder, (E) fat tissue, (F) eyes, (G) muscles, (H) thyroid, (I) trachea, (J) stomach and gastroliths, (K) lungs, (L) liver, (M) heart and (N) small intestines.

Briefly, crocodile blood was collected in sterile ethylenediaminetetraacetic acid (K_2_EDTA) vacutainers (Becton Dickinson, Franklin Lakes, New Jersey, USA) and the internal organs were dissected out using aseptic techniques by a pathologist of CITES-registered crocodile farm. A female crocodile, weighing 25 kg, measuring 193 cm in length and 43 cm in width was caught, physically restrained and the blood was collected using a 26-gauge needle from the supravertebral vein,^16^ post-occipital venous sinus^17^ on the head and from the ventral coccygeal vein that is located on the tail region.^18^ Additionally, internal organs were collected by making an incision along the ventral midline, from the cloaca until the lower jaw.^19^ The internal organs were collected in sterile containers (figure 2). These procedures are routinely carried out by the personnel at the farm, and were conducted by these professionals.

### Preparation of organ lysates and serum

Briefly, crocodile blood was centrifuged at 3000 × g for 15 min at 37°C.^20 21^ Next, the supernatant was collected and stored at −80°C in aliquots until tested further.^4^ For the preparation of crude organ lysates, the internal organs were cut to small pieces and homogenised using mortar and pestle^22^ with sterile distilled water as the solvent. Of note, 10 µL/mL of protease inhibitors and EDTA was then added to the lysates. Subsequently, lysates were subjected to 10 rounds of freeze-thaw, sonicated on ice for approximately 3 min at 20 kHz using a Cole–Parmer Ultrasonic Processor and centrifugated at 20 000 × g for 80 min at 4°C.^4 23^ Next, the supernatants were collected, filtered using sterile 0.2 µm pore-sized syringe filters (Sartorius, Germany) and stored at −80°C in aliquots until tested further.^4^ The protein concentration of crude organ lysates and serum was determined via Bradford assay.^4^

### Culture of cell lines

Human cervical adenocarcinoma cells (HeLa) (American Type Cell Culture (ATCC)CCL-2), human breast adenocarcinoma cells (MCF7) (ATCCHTB-22) and human prostate adenocarcinoma cells (PC3) (ATCCCRL-1435) were obtained from ATCC. Human keratinocyte skin cells (Hacat) (CVCL_0038 and Cell Lines Service (CLS): 300493) were procured from CLS, Germany. Briefly, cells were cultivated in Roswell Park Memorial Institute-1640 supplemented with 10% (v/v) FBS, 1% L-glutamine, 1% MEM NEAA and 1% of antibiotic solution containing 10 000 U/mL of penicillin and 10 000 µg/mL streptomycin. The cells were incubated at 37°C in a 95% humidified incubator with 5% carbon dioxide (CO_2_).^4 24^

### Growth inhibition assay

A growth inhibition assay was done to investigate the ability of organ lysates and serum to inhibit the growth of cancer cells. Briefly, 3×10^4^ cells were cultured onto 96-well plates and incubated at 37°C in a 95% humidified incubator with 5% CO_2_ until an approximately 50% semiconfluent monolayer of cells was achieved. Next, media were removed and cells were treated with 100 µg/mL of organ lysates and 10% (v/v) of sera for 24 hours. An initial cell count was performed on the control well to determine the number of cells present in the monolayer of the 50% semiconfluent well. Once a 100% confluent monolayer of cells were achieved in the control well, the media was discarded and the cells were detached by incubation with 2.5 g/L trypsin solution for 5 min at 37°C. Next, fresh media supplemented with 10% (v/v) FBS was added to stop trypsin activity and cells were centrifuged at 3000 × g for 5 min. Supernatant was then discarded, the cell pellet was resuspended in fresh media and viable cells were counted using Trypan blue exclusion assay. The membrane of cells treated with organ lysates and serum/haemolymph, if damaged, enabled penetration of Trypan blue dye. As a result, these damaged and non-viable cells were stained blue. In contrast, intact membrane of viable cells prevented the penetration of Trypan blue dye, resulting in unstained cells.^25^ The percentage of cell growth was calculated by comparing the number of viable cells present in treated wells and control wells containing 10% (v/v) FBS (control for serum). The growth inhibition was calculated as follows: Total cells per mL=Total cells counted × [dilution factor/number of counted squares] × 10 000 cells per mL. The data are representative of the mean±SE of at least three independent experiments performed in duplicate.

### Cell cytotoxicity assay

Cell cytotoxicity assay was done to investigate the cytotoxic effects of organ lysates and serum against cancer cells. Briefly, 4×10^5^ cells were cultivated in 96-well plates and incubated overnight to achieve confluent monolayers. Next, media was replaced and cells were treated with 100 µg/mL of organ lysates and 10% of serum for 24 hours at 37°C in a 5% CO_2_ incubator. The negative control wells were treated with media alone and FBS (control for serum). After 24 hours, the supernatant of each well was collected and percentage cell death was determined using LDH cytotoxicity detection kit.^4^ LDH is a soluble cytosolic enzyme which is present in all viable cells. Cells with affected and damaged membrane permeability lead to the release of LDH enzymes from the cytoplasm to the surrounding matrix. The cell supernatant containing LDH enzyme catalyses the conversion of lactate to pyruvate, resulting in the generation of NADH and H^+^. Following that, the diaphorase enzyme (catalyst solution from the kit) transfers the H and H^+^ from NADH and H^+^ to the tetrazolium salt p-iodonitrotetrazolium violet (solution in the kit), resulting in the reduction of this colourless salt to the red formazan dye. The absorbance of each well was then measured via a microplate reader at 490 nm. The positive control well that represents total cell death was prepared by incubating the cells with 0.1% Triton X-100 at 37°C for 60 min. The percentage cell death was determined as follows: test value – control value/total LDH release – control value × 100 = %cytotoxicity. The data are representative of the mean±SE of at least three independent experiments performed in duplicate.

### Cell survival assay

Cell survival assay was done to investigate the revival potential of cancer cells treated with organ lysates and serum. Briefly, cells were grown to confluency in 96 plates. Next, media was removed and the cells were treated with 100 µg/mL and 10% of serum for 24 hours. The negative control wells were treated with media alone and FBS (control for serum). Next, the supernatant was discarded and the cells were detached using 2.5 g/L trypsin for 5 min. Media supplemented with 10% (v/v) FBS was then added to stop the activity of trypsin and the cell suspension was subjected to centrifugation at 3000 × g for 5 min. The cell pellet was then resuspended with fresh media and regrown in 96-well plates. After 24 hours, the number of cells were enumerated to determine cell growth. The data are representative of the mean±SE of at least three independent experiments performed in duplicate.

### Heat inactivation of crocodile serum

To investigate the nature of potential anticancer molecules from crocodile serum, the serum was heat inactivated by boiling at 56°C and 65°C for 30 min and 99°C for 5 min^26–28^ and cooling at 4°C. HeLa cells were then incubated with 10% (v/v) heat-treated serum for 24 hours at 37°C in a 5% CO_2_ incubator and subjected to LDH cytotoxicity assay as described previously.^4^ The data are representative of the mean±SE of at least three independent experiments performed in duplicate.

### Crocodile serum protein digestion using pronase enzyme

Crocodile serum was treated with 7U of pronase enzyme that was prepared as a stock solution of 1 mg/mL for 1 hour at 37°C in a 5% CO_2_ incubator. Next, HeLa cells were treated with 10% (v/v) of pronase-treated serum at 37°C for 24 hours in a 5% CO_2_ incubator. These cells were then subjected to LDH cytotoxicity assay as previously described.^4^ Negative control wells consisted of HeLa cells treated with fresh media alone and pronase-treated FBS. The data are representative of the mean±SE of at least three independent experiments performed in duplicate.

### Liquid chromatography mass spectrometry analysis of small molecules in sera samples

Liquid–liquid extraction method using a mixture of ice-cold methanol and ultra-pure deionised MiLi-Q water was performed on the samples.^29^ Briefly, 800 µL of ice-cold mixture containing HPLC-grade methanol and ultra-pure deionised MiLi-Q water at a ratio of (8:1) (v/v) was mixed with 100 µL of serum, vortexed for 2 min and stored at 4°C. After 30 min, sample was subjected to centrifugation at 7000 × g for 8 min, and supernatant was collected, filtered and stored at −80°C. Liquid chromatography mass spectrometry (LC-MS) analyses were done using Agilent 1290 infinity liquid chromatography (LC) system that was linked to Agilent 6520 accurate-mass quadrupole–time of flight (Q-TOF) mass spectrometer with dual electrospray ionisation (ESI) source (Agilent Technologies, Santa Clara, California, USA). Briefly, 1.0 µL of sample was injected and the chromatographic separation was achieved using Agilent Zorbax Eclipse XDB-C18, narrow bore 2.1×150 mm, 3.5 μm (P/N: 9 30 990–902) column. The column temperature was maintained at 25°C and the samples were housed in an autosampler at 4°C. Solvent A (0.1% formic acid in water) and solvent B (0.1% formic acid in acetonitrile) with a flow rate of 0.5 mL/min were used for the mobile phase. Nitrogen gas (carrier gas) had a flow rate of 10 L/min and temperature that was set at 300°C. For mass spectrometry (MS), LC eluents were analysed with an Agilent 6520 Accurate-Mass Q-TOF mass spectrometer with ESI source in positive and negative ionisation mode. The mass range was set to 100–3200 m/z using both negative and positive ionisation mode. The fragmentation voltage was set at 125 V and the capillary voltage was set at 4000 V for positive ionisation mode and 3500 V for negative ionisation mode. The raw LC-MS data were acquired and processed using the Molecular Feature Extraction in Agilent Mass Hunter Qualitative Analysis B.05.00 software. Only peaks with abundance value of above 5000 counts with a relative height of more than 2.5% were selected. The small molecules were identified using the METLIN Personal Metabolite Database and Molecular Formula Generation software (Agilent Technologies). The data are representative of the mean±SE of at least three independent experiments performed in duplicate.

### Identification of potential anticancer peptides

Protein samples were digested as described previously using *In-solution* digestion method.^30^ Approximately 1 mg/mL of serum protein was mixed with 50 µL of 100 mM ammonium bicarbonate, 50 µL of trifluoroethanol and 2 µL of 200 mM dithiothreitol, vortexed and incubated for 1 hour at 60°C. To alkylate the proteins, 8 µL of 200 mM iodoacetamide was added and incubated for 1 hour in the dark at room temperature. Next, 2 µL of 200 mM dithiothreitol was added into the mixture and incubated for 1 hour in the dark for the removal of excess iodoacetamide. The pH of the mixture was adjusted to pH 7–9 using 600 µL of distilled water and 200 µL of 100 mM ammonium bicarbonate. Next, 50 µL of 2.5 g/L trypsin was added to the protein mixture and incubated overnight at 37°C. To deactivate trypsin activity, 1 µL of formic acid was added and the mixture was stored at −80°C before analysis. The samples were subjected to LC-MS/MS (iquid chromatography–mass spectrometry (LC-MS)) analyses using Agilent 1200 HPLC-Chip/MS Interface, coupled with Agilent 6550 iFunnel Q-TOF LC/MS. Briefly, 1.0 µL of digested sample was injected and separation of molecules was achieved using Agilent Large Capacity Chip, 300 Å, C18, 160 nL enrichment column and 75 μm × 150 mm analytical column (P/N: G4240-62010) which was equilibrated with 0.1% formic acid in water (solvent A). The samples were housed in an autosampler at 4°C and eluted via an increasing gradient of 90% acetonitrile in 0.1% formic acid in water (solvent B) as follows: 5%–75% solution B from 0 to 30 min and 75% from 30 to 39 min with a flow rate of 4 μL/min from Agilent 1200 Series capillary pump and 0.5 μL/min from Agilent 1200 Series nano pump. Nitrogen gas (carrier gas) had a flow rate of 5 L/min and temperature that was set at 325°C. For MS, LC eluents were analysed with an Agilent 6550 iFunnel Q-TOF LC-MS/MS in positive ionisation mode. The protein spectrum mass range was set to 110–3000 m/z for MS and 50–3000 m/z for MS/MS scan. The fragmentation voltage was set at 360 V and the capillary voltage was set at 2050 V. The mass spectrum analysis was achieved with Agilent Mass Hunter data acquisition software and PEAKS V.7.0 software.

### Serum peptide detection and protein identification by automated de novo sequencing

From the list of peptides detected, automated de novo sequencing was performed using PEAKS Studio V.7.0 (Bioinformatics Solution, Waterloo, Canada). For protein identification and homology search by de novo sequence comparison, SwissProt.Crocodylia (January 2019) database was used for crocodile (*C. porosus*). Enzyme used for protein digestion was set as trypsin and the false discovery rate of 1% and ≥2 unique peptides were set as parameters to exclude inaccurate proteins. A −10lgP score of greater than 20 was set to indicate proteins with high confidence scores. The presence of potential anticancer peptides (ACP) was predicted using the Machine-Learning-Based Prediction of Anticancer Peptides (MLACP) tool.^31^

### Differential gene expression analysis of cancer cells treated with crocodile sera

Briefly, cells grown to confluency were treated with 10% of serum for 40 min. The control well cells were treated with FBS. Next, the supernatant was discarded and the cells were detached using 2.5 g/L trypsin for 5 min. Phosphate-buffered saline supplemented with 10% (v/v) FBS was then added to stop trypsin activity and cell suspension was subjected to centrifugation at 300 × g for 5 min. The supernatant was discarded and RNA was extracted from the treated cells as per manufacturer’s guidelines. The cell pellet was resuspended with 700 µL of Qiazol cell lysis buffer, vortexed, homogenised using syringe and needle and incubated at room temperature for 5 min. Next, 140 µL chloroform were added and the sample was mixed by inverting the tubes for 15 s followed by incubation at room temperature for 3 min for the separation of aqueous and organic phase. The sample was then centrifuged at 12 000×g for 15 min at 4°C and the RNAs on the uppermost layer (aqueous phase) were collected. The sample was then washed, purified and eluted using the buffers provided in the kit as per manufacturer’s guidelines (Qiagen miRNeasy Kit, Canada). RNA concentration and quality were assessed using BioDrop Duo Micro-Volume UV–Vis Spectrophotometer. Pure RNA samples should comprise of a ratio between 1.8 and 2.0 when measured at the spectrophotometric relative absorbance ratio at (260 nm/280 nm). RNA quality was then further assessed using Agilent Bioanalyzer for RNA integrity number (≥ 6.5) score.^32^ Sequencing libraries were then prepared using the NEBNext Ultra RNA Library Prep Kit for Illumina (New England Biolabs, Ipswich, Massachusetts, USA) following manufacturer’s instructions. The size quality of libraries was assessed using the Bioanalyzer 2100 High Sensitivity DNA chip (Agilent Technologies, Waldbronn, Germany) and subjected to Next Generation Sequencing using Miseq System (Illumina, San Diego, California, USA). The differential gene expression of treated cells was then analysed using Galaxy online tool (Galaxy, Baltimore, Maryland, USA). Briefly, the generated sequences were trimmed using the Trimmomatic tool to remove the adapters and noisy background. Next, sequences were aligned with the human reference genome (*Homosappien*; GRCh38) using RNAstar followed by the quantification of gene expression using the FeatureCounts tool from Galaxy online tool. The differential gene expression was then done using DeSeq2 which calculated the significance of the expressed genes using Wald statistical method (Galaxy, USA). DESeq2 performs normalisation for each gene across samples to correct for any library size and RNA composition bias such that a small number of genes are very highly expressed in one experiment condition but not in the other. DESeq2 uses shrinkage estimation for dispersions and fold changes. DESeq2 uses the Wald test for significance and Benjamini–Hochberg adjustment for multiple testing problems.^33^ The data are representative of the mean±SE of at least three independent experiments performed in duplicate.

## Results

### Sera and organ lysates of crocodile demonstrated irreversible growth inhibition and cytotoxic activity against cancer cells

**Figure 3:**
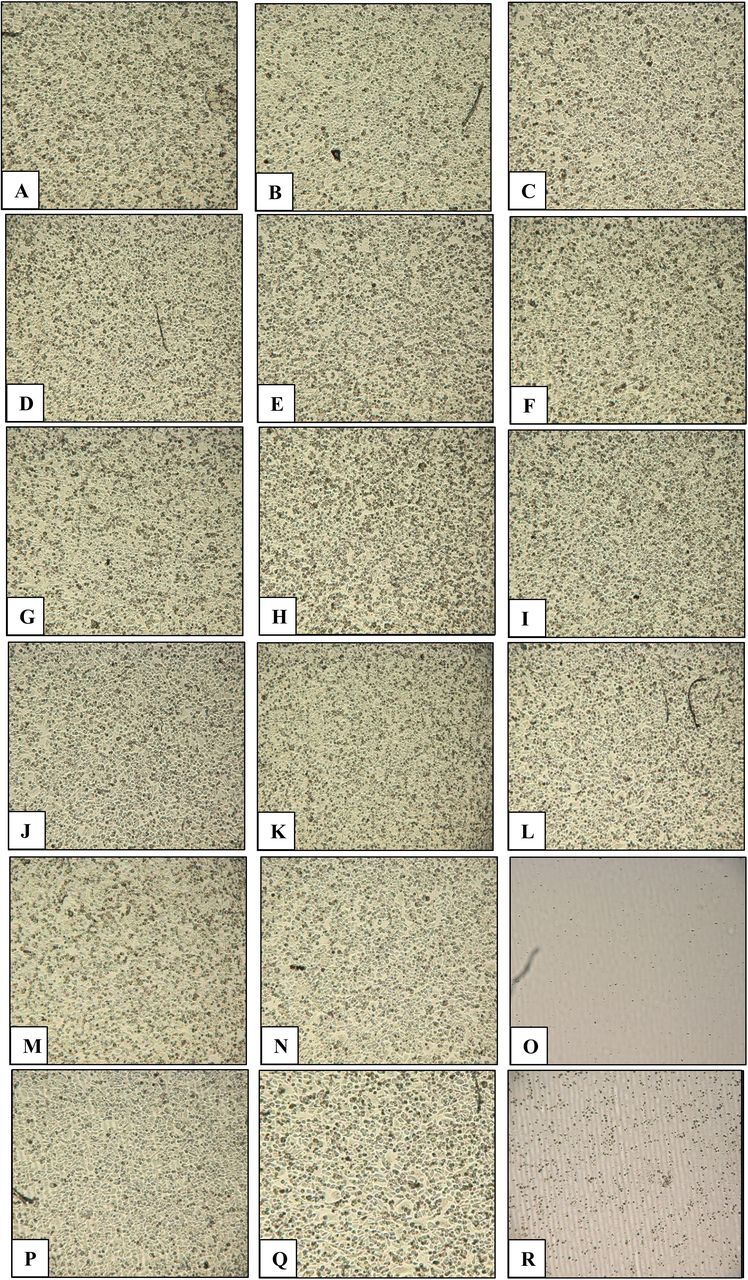
(A–R) Cell survival ability of HeLa cells treated with lysates of crocodile (*Crocodylus porosus*). Representative images of HeLa cells with and without crude lysates (A: control; B: bovine serum albumin; C: fetal bovine serum; D: fat water; E: fat; F: liver; G: tail muscles; H: stomach; I: thyroid; J: eyes; K: heart; L: trachea; M: lungs; N: large intestines; O: gall bladder; P: small intestines; Q: kidneys and R: serum).

**Figure 4:**
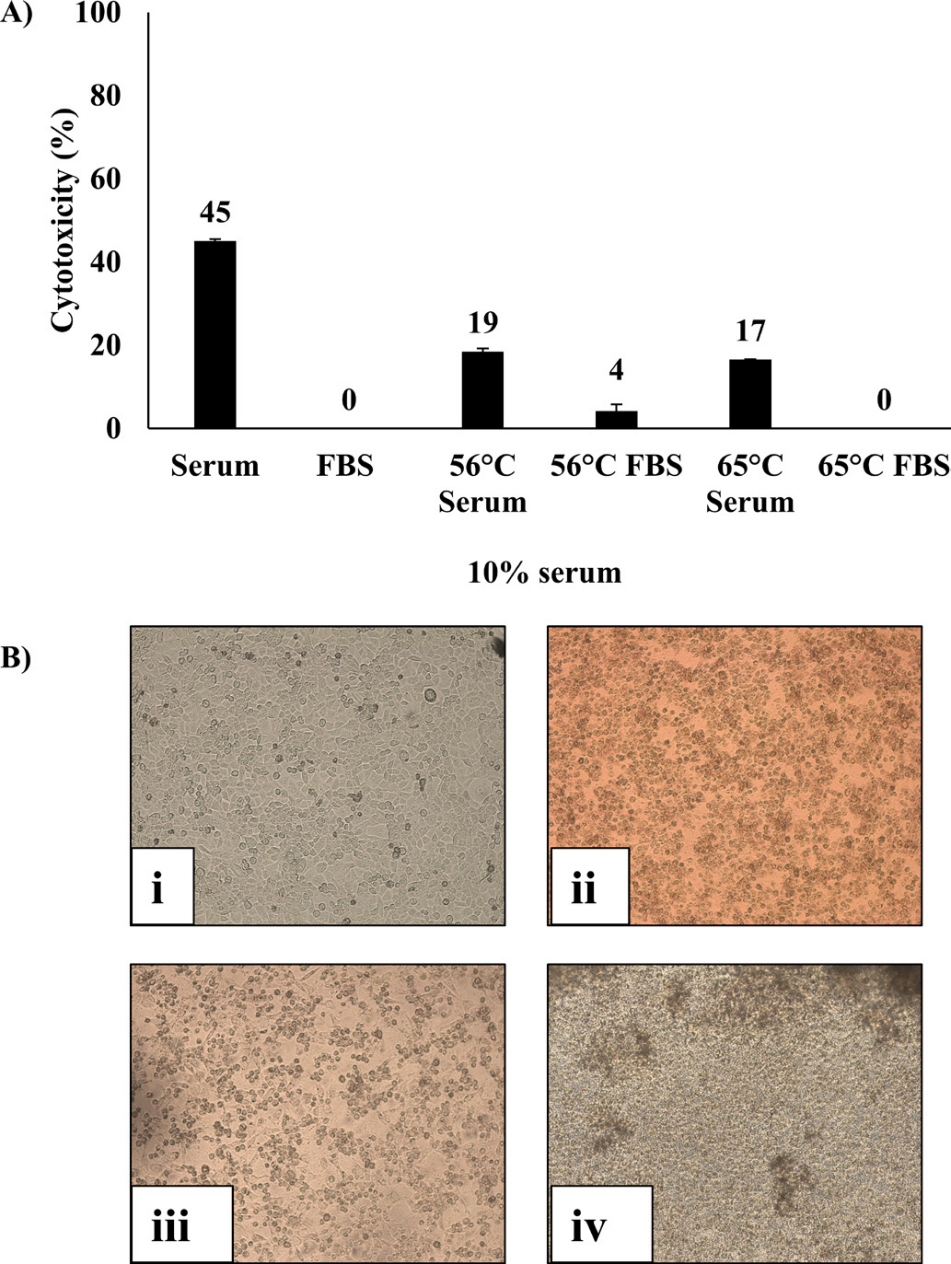
(A–B) The cytotoxic effects of heat-inactivated crocodile (*Crocodylus porosus*) serum (100× magnification). Briefly, HeLa cells were incubated with 10% crude unboiled, 56°C and 65°C boiled serum at 37°C for 24 hours. Next day, cell cytotoxicity assay was performed by determining the concentration of lactate dehydrogenase enzyme released by the affected cells at 490 nm. Representative images of HeLa cells treated with boiled and unboiled serum and FBS control. (i: FBS control; ii: unboiled serum; iii: serum boiled at 56°C; iv: serum boiled at 65°C). FBS, fetal bovine serum.

**Figure 5:**
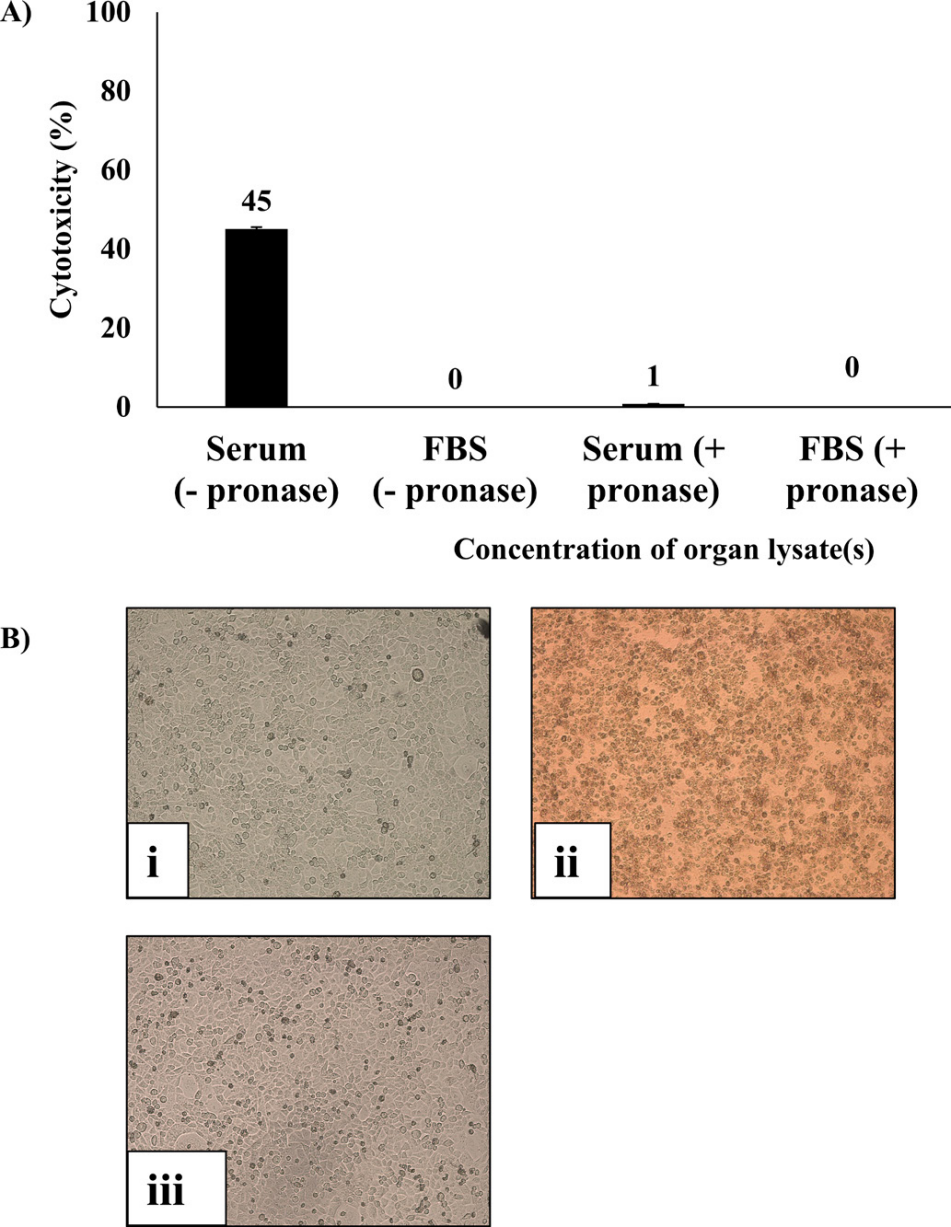
(A–B) The cytotoxic effects of pronase-treated crocodile (*Crocodylus porosus*) serum (100× magnification). Briefly, HeLa cells were incubated with serum and pronase-treated serum at 37°C for 24 hours. Next day, cell cytotoxicity assay was performed by determining the concentration of lactate dehydrogenase enzyme released by the affected cells at 490 nm. Representative images of HeLa cells treated with serum and pronase-treated serum (i: FBS control; ii: serum; iii: pronase-treated serum). FBS, fetal bovine serum.

Among various lysates, gall bladder lysate and sera of *C. porosus* but not bovine exhibited growth inhibition and cytotoxic effects against cancer cells (p<0.05 using independent t-test, two-tailed distribution) (table 1). Among other lysates, growth inhibition and cytotoxic effects are selective against HeLa cells, PC3 cells and MCF7 cells. *C. porosus* gall bladder lysates containing gall fluid and 10% serum inhibited more than 99% growth of HeLa cells, PC3 cells and MCF7 cells (p<0.05), whereas the gall bladder lysate was cytotoxic against HeLa cells, PC3 cells and MCF7 cells (p<0.05) (table 1). The serum of *C. porosus* was cytotoxic against HeLa cells and MCF7 cells (p<0.05) (table 1). Most importantly, cell survival ability of HeLa cervical adenocarcinoma cells treated with 100 µg/mL of crude lysate and 10% (v/v) of serum revealed that serum and gall bladder lysate of *C. porosus* exhibited irreversible killing effect against HeLa cells as compared with the control (figure 3). Heat-treated *C. porosus* serum demonstrated reduced cytotoxic effects against HeLa cells (figure 4) whereas pronase-digested serum did not demonstrate cytotoxic effects against HeLa cells (figure 5).

**Table 1:** The growth and cytotoxic effects of serum and organ lysates of crocodile (*Crocodylus porosus*) against human cancer and normal cells

Animal species	Lysates/ serum	Growth (%)			Cytotoxicity (%)		
		HeLa	PC3	MCF7	HeLa	PC3	MCF7
**Crocodile** (***C. porosus***)	**Fat water**	100	98±2.39	100	1±1.25	18±1.69	0
	**Fat**	100	100	97±2.84	0	17±2.22	0
	**Liver**	87±8.68	100	88±12.30	40±1.51*	28±0.29*	0
	**Tail muscles**	100	100	96±0.39	12±1.47	17±1.08	0
	**Stomach**	100	100	94±0.58	19±1.04*	0	0
	**Thyroid**	0*	30±9.55	61±8.80	29±0.86*	17±5.26	0
	**Eyes**	100	100	98±1.89	36±7.61	19±0.32	0
	**Heart**	100	100	96±3.78	37±0.03*	27±1.59*	0
	**Trachea**	100	100	100	0	3±2.71	0
	**Lungs**	100	99±0.80	100	13±2.87	6±0.35	0
	**Large intestines**	100	100	100	1±1.26	6±0.32	0
	**Gall bladder**	0*	0*	0*	49±0.38*	21±1.71*	62±2.90*
	**Small intestines**	100	100	100	0	0	0
	**Kidneys**	0*	0*	70±13.78	0	0	0
	**Serum**	0*	0*	0*	55±0.55*	56±5.83	50±1.57*

Briefly, semiconfluent cells were incubated with 100 µg/mL of lysates and 10% serum and growth inhibitory effects were determined. For cytotoxicity assessment, confluent cells were incubated with lysates and serum. Note that serum, gall bladder and thyroid of *C. porosus* showed growth inhibition as well as cytotoxicity against cancer cells (p<0.05 using independent t-test, two-tailed distribution). Asterisk and bold denote significant (p<0.05) difference. Data are presented as the mean±SE of at least three independent experiments performed in duplicates.

### Potential anticancer small molecules and potential ACP were identified from crocodile sera

Serum is made up of approximately 95% of water content, therefore a polar extraction solvent (methanol) was used for the extraction of small molecules^29^ and analysed using LC-MS. A total of 80 small molecules were detected and a total of 19 small molecules were putatively identified from the serum of the saltwater *C. porosus* when compared and matched against the METLIN metabolomics database (table 2). It was reported that lesser than 24 small molecules are normally detected and identified at a time due to the limitation in the availability of metabolite databases.^29^ Using LC-MS/MS approach as described earlier, the serum of *C. porosus* demonstrated seven peptides belonging to the alpha-2-macroglobulin isoform X2 (Accession ID: A0A1U7S0T0) protein family, five peptides belonging to the transferrin (Accession ID: A0A286T2Q9) protein family, six peptides belonging to the Ccmplement C3 (Accession ID: A0A1U7S0C0) protein family, three peptides belonging to the fibrinogen beta chain (Accession ID: A0A1U7SP96) protein family, two peptides belonging to the haemoglobin subunit beta (Accession ID: P86919) protein family and three peptides belonging to the serum albumin isoform X2 (Accession ID: A0A1U8CYA2) protein family (table 3). A total of 207 peptides were predicted to be potential ACP from a total of 749 detected using LC-MS/MS (table 4).

**Table 2:** Compounds identified from the serum of crocodile (*Crocodylus porosus*) via liquid chromatography mass spectrometry

	Compound	Mass	m/z	Reported activity
**Compounds detected: 80; compounds identified: 19**				
**1**	2-Amino-3-methyl-1-butanol Molecular formula: C5 H13 N O CAS: 473-75-6 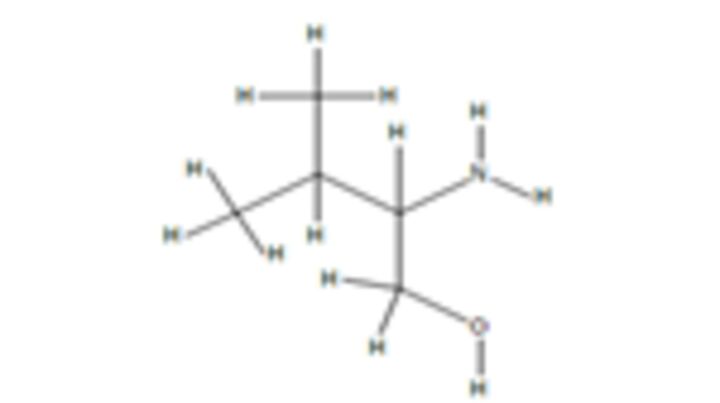	103.10	104.11	Derivatives of imidazolines-oxazolines prepared from acid derivatives and enantiomerically pure (S)−2-amino-3-methyl-1-butanol exhibited antibacterial and antifungal activity^49^
**2**	4-Methylaminobutyrate Molecular formula: C5 H11 N O2 CAS: 1119-48-8 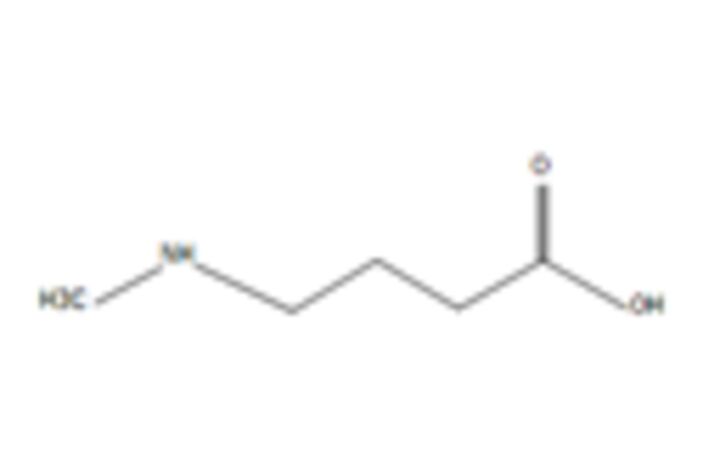	117.08	118.09	No reported activity
**3**	Isoamyl nitrite Molecular formula: C5 H11 N O2 CAS: NA 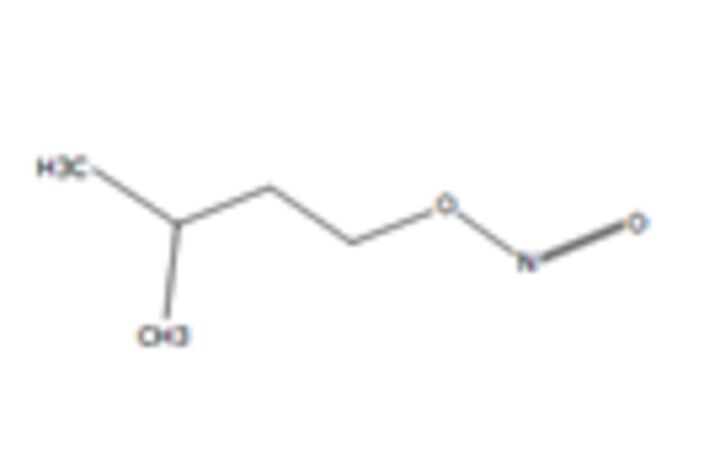	117.08	118.09	No reported activity
**4**	Purine Molecular formula: C5 H4 N4 CAS: 120-73-0 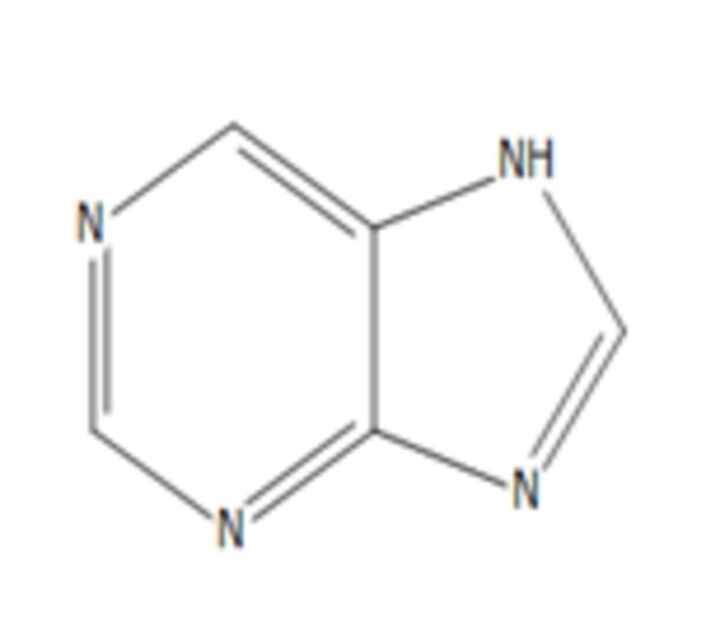	120.04	121.05	Purine derivatives such as 2,6-dipropynylthio-7-methylpurine 4, 2-chloro-6,8-dipropynylthio-7-methylpurine 14, and 2-chloro-6,8-di(N-morpholinylbutynylthio)-7-methylpurine exhibited anticancer activity against glioblastoma SNB-19, melanoma C-32 and adenocarcinoma MDA-MB-231^50^ Novel derivatives of purine analogues were found to possess anticancer activities against various cancer cell lines^41^
**5**	Creatine Molecular formula: C4 H9 N3 O2 CAS: 57-00-1 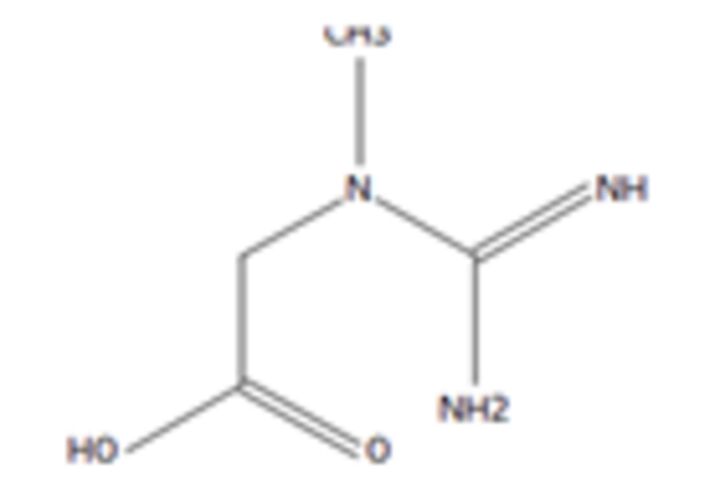	131.07	132.08	Used as a supplement along with potential anticancer compounds to inhibit and slow down the proliferation rate or growth of Walker 256 tumour cells in rats with no toxicity^51^ Reduces the degree of nephrotoxicity effect due to anticancer drugs such as cisplatin^52^ (Boc)2-creatine compound that has creatine exhibits anticancer activity against cancer cells via blocking creatine kinase activity^53^
**6**	Aminocaproic acid Molecular formula: C6 H13 N O2 CAS: 60-32-2 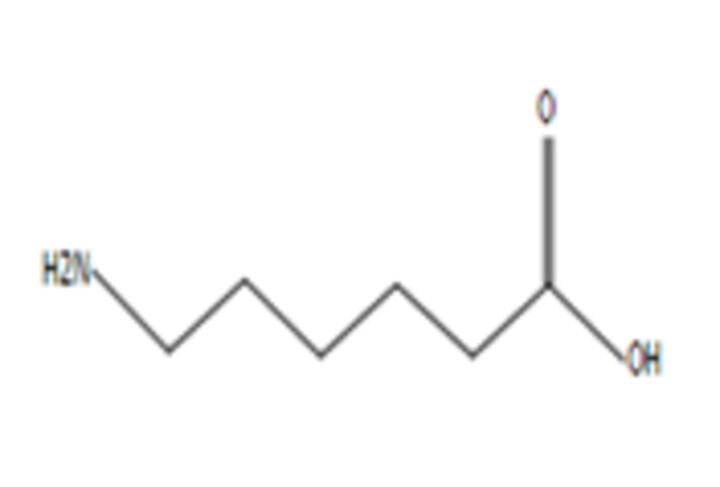	131.09	132.10	NA
**7**	N-Acryloylglycine Molecular formula: C5 H7 N O3 CAS: 24599-25-5 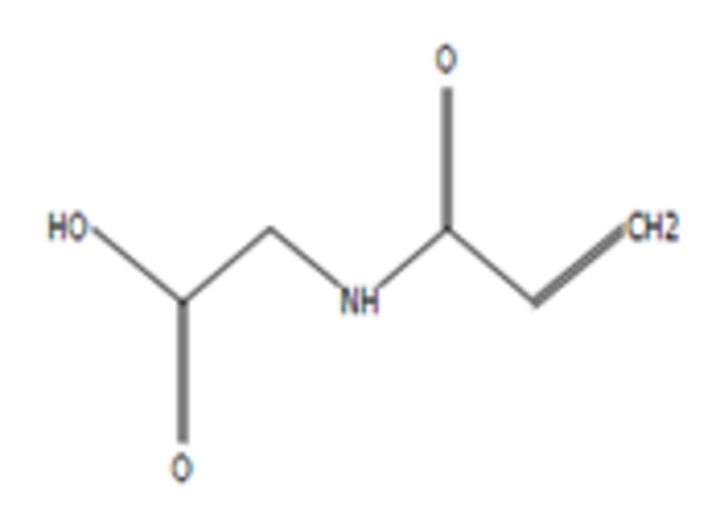	129.04	147.08	Drug carrier^54^
**8**	L-Methionine Molecular formula: C5 H11 N O2 S CAS: 63-68-3 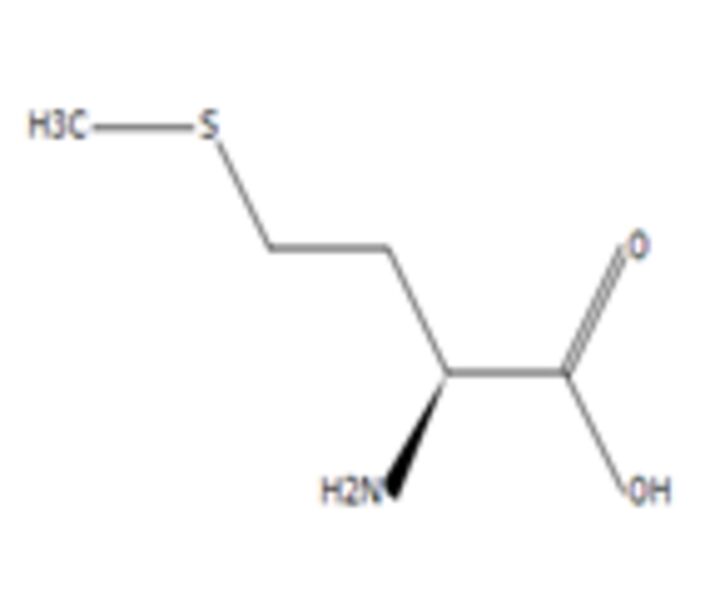	149.05	150.06	Inhibits growth of cancer cells by disrupting the cell cycle of BXPC-3 (mutated p53) and HPAC (wild-type p53) pancreatic cancer cells besides triggering apoptosis mechanism in BXPC-3 pancreatic cancer cells^55^
**9**	Erythronic acid Molecular formula: C4 H8 O5 CAS: NA 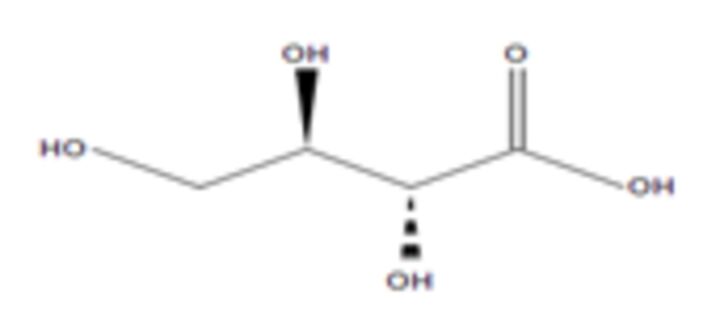	136.04	159.03	Urinary metabolites such as erythronic acid, 5-oxoprolinate and N-acetylaspartic acid was highly elevated in Human papillomavirus (HPV) patients as compared with negative controls suggesting that this metabolite can be used as a marker for high-risk HPV-infected patients^56^
**10**	Benzocaine Molecular formula: C9 H11 N O2 CAS: 94-09-7 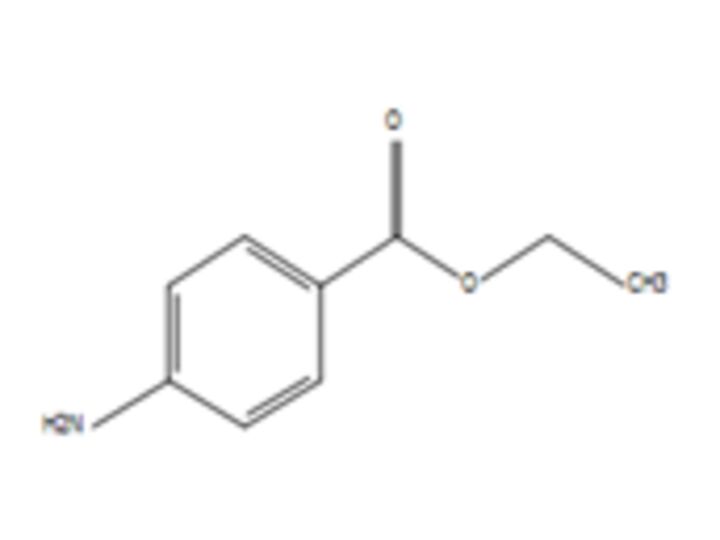	165.08	166.09	A type of local anaesthetic that can induce methaemoglobinaemia^57^
**11**	N-alpha-Methylhistidine Molecular formula: C7 H11 N3 O2 CAS: 24886-03-1 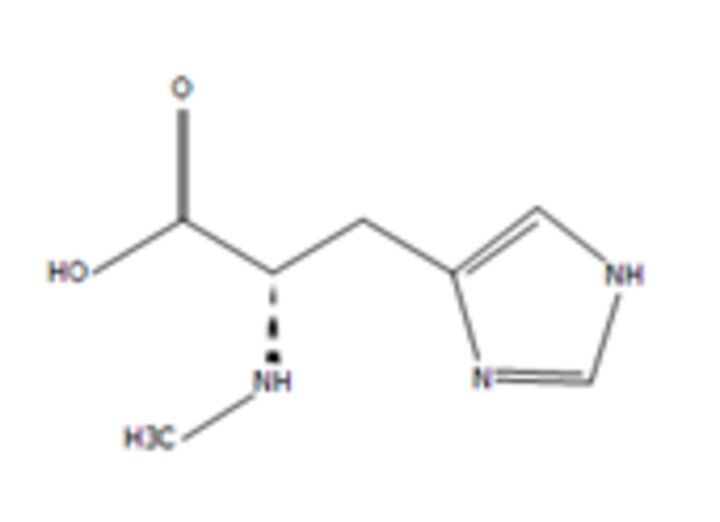	169.08	170.09	NA
**12**	Corchorifatty acid A Molecular formula: C18 H28 O4 CAS: NA 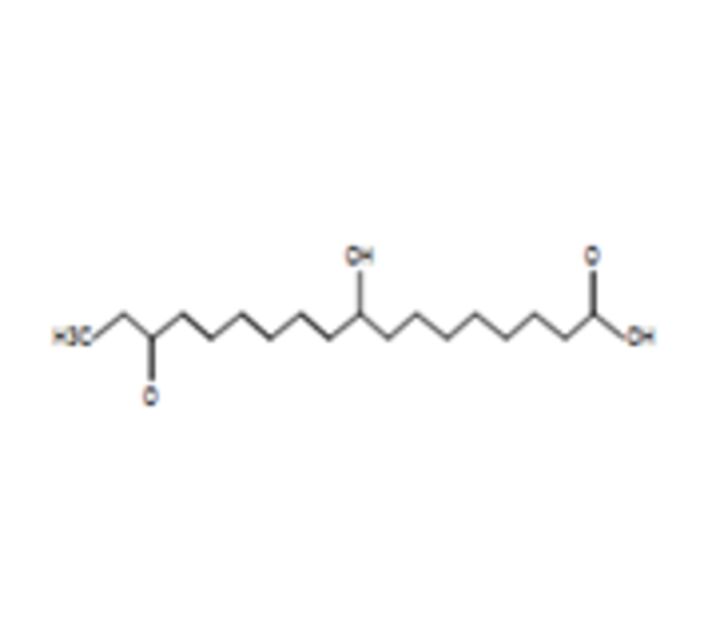	308.20	309.20	A type of corchorifatty acid known as corchorifatty acid B that was isolated from ethanol extracts of the aerial parts of *Melissa ofﬁcinalis* Linne’ (Labiatae) exhibited inhibitory effects on cellular pigmentation/ melanogenesis^58^
**13**	Artocarpin Molecular formula: C26 H28 O6 CAS: NA 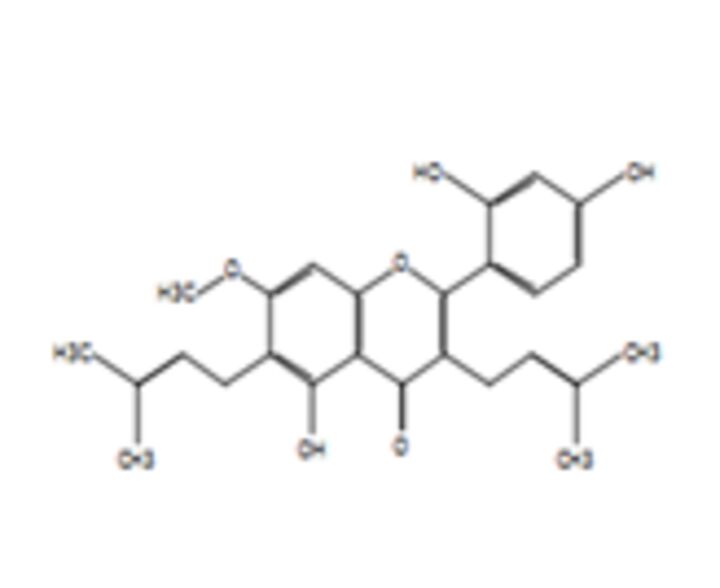	436.19	437.19	Artocarpin exhibits would healing activity^59^ Artocarpin selectively was cytotoxic against human colon cancer cells via resulting in cell cycle arrest and inducing apoptosis^60^
**14**	Uric acid Molecular formula: C5 H4 N4 O3 CAS: 69-93-2 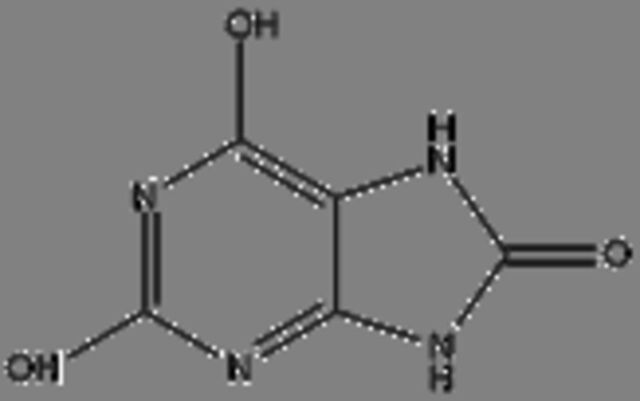	168.03	167.02	Demonstrates antioxidative effects in terms of being a strong peroxyl radical, hydroxyl radicals and singlet oxygen scavenger that may have a positive effect against cancer and ageing process^61^ Uric acid acts as an antioxidant, provides neuroprotection and activates immune and inflammatory responses^62^ Uric acid has oxidative and antioxidative properties^63^ Uric acid plays a role in the prolonged lifespans of termites^64^ Uric acid has antioxidative effects against neurodegenerative disease^65^
**15**	2-Hydroxyethanesulfonate Molecular formula: C2 H6 O4 S CAS: 107-36-8 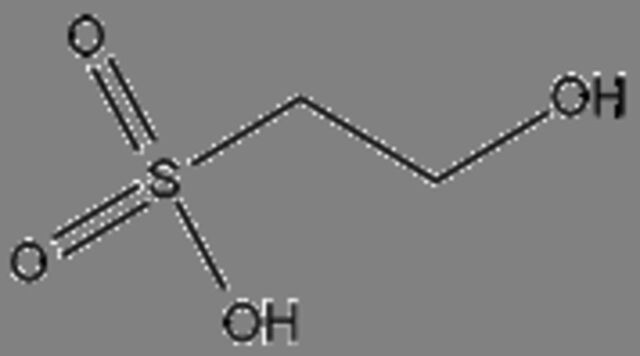	126.00	185.01	2-Hydroxyethanesulfonate, a component in the drug NSC 370147, exhibited more effective anticancer activity against multidrug-resistant cells as compared with vincristine^66^ 2-Hydroxyethanesulfonate, a component in the drug NSC 370147, prevents drug resistance murine P388 murine tumour cells when treated in combination with doxorubicin, melphalan, cisplatin or methotrexaten^67^
**16**	2-(Fluoromethoxy)-1,1,3,3,3-pentafluoro-1-propene (compound A) Molecular formula: C4 H2 F6 O CAS: 58109-34-5 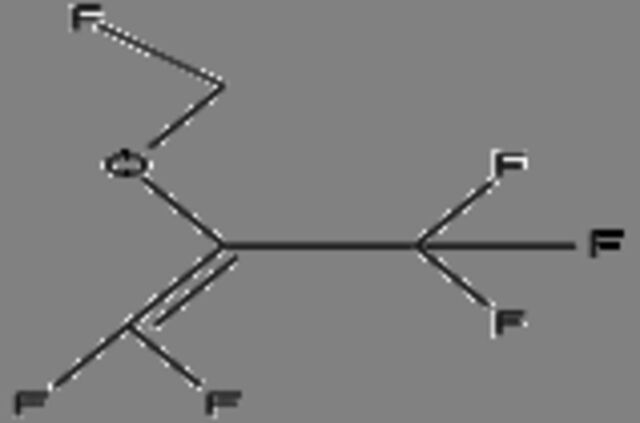	180.00	225.00	No reported activity
**17**	**Valdecoxib** Molecular formula: C16 H14 N2 O3 S CAS: 181695-72-7 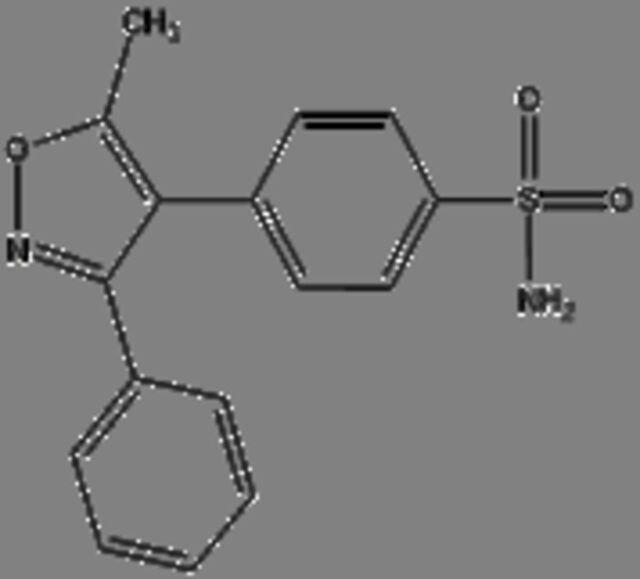	314.07	315.08	COX-2 inhibitor to treat inflammation in rheumatoid arthritis patients^40^ Used to treat moderate to severe osteoarthritis of the knee^68^ Pain killer for menstrual pain due to primary dysmenorrhoea (cramps in the lower abdomen before or during menstruation)^69^ Altered the lipid composition of cell membrane which resulted in anti-inflammatory activity in cancer cells^38^
**18**	Rofecoxib Molecular formula: C17 H14 O4 S CAS: 162011-90-7 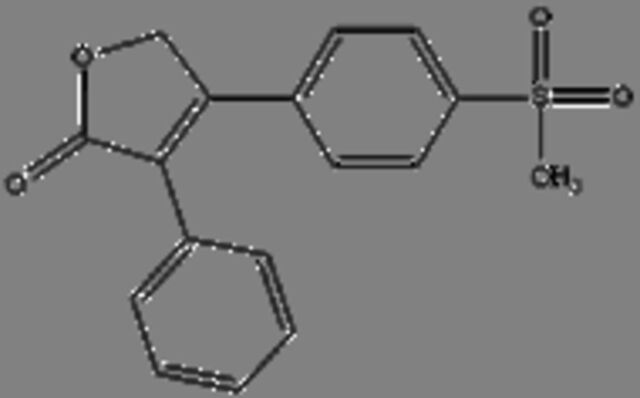	314.06	313.05	Rofecoxib, a COX-2 inhibitor exhibited anticancer effects against BGC-823 independently and in combination with other anticancer drugs^70^ Combination of cyclophosphamide, vinblastine and rofecoxib exhibited anticancer activity in patients with advanced solid tumours with minimal side effects^71^ Rofecoxib protects against UVB-induced DNA damage via mechanisms not related to the inhibition COX-2^72^
**19**	Pseudobaptigenin 7-O-laminaribioside Molecular formula: C28 H30 O15 CAS: NA 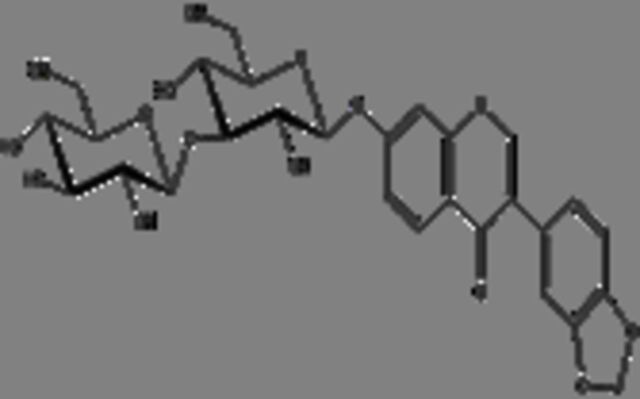	606.16	605.15	NA

COX-2, cyclooxygenase 2.

**Table 3:** List of detected proteins from *in-solution* digested serum of crocodile (*Crocodylus porosus*) via LC-MS/MS

Accession ID	−10lgP	Coverage (%)	#Peptide	#Unique peptide	Description
A0A1U7S0T0|A0A1U7S0T0_ALLSI	115.10	4	8	7	alpha-2-macroglobulin isoform X2 (*Alligator sinensis*)
A0A286T2Q9|A0A286T2Q9_CROSI	106.55	4	5	5	Transferrin (*Crocodylus siamensis*)
A0A1U7S0C0|A0A1U7S0C0_ALLSI	98.45	3	7	6	Complement C3 (*A. sinensis*)
A0A1U7SP96|A0A1U7SP96_ALLSI	91.00	7	3	3	Fibrinogen beta chain (*A. sinensis*)
P86919|HBB_CROSI	80.86	12	2	2	Haemoglobin subunit beta (*C. siamensis*)
A0A1U8CYA2|A0A1U8CYA2_ALLSI	69.59	6	3	3	Serum albumin isoform X2 (*A. sinensis*)

**Table 4:** Potential anticancer peptides from the serum of crocodile (*Crocodylus porosus*) predicted using MLACP tool

No.	Peptide sequence	Length	RFACP (probability)		SVMACP (probability)	
1	FKMWPSSPAVPLAPK	15	ACP	0.5416	ACP	0.5518
2	WFDDKHFGLPPKER	14	ACP	0.5583	Non-ACP	0.4127
3	LLNPMLPDPPLPK	13	Non-ACP	0.4048	ACP	0.5497
4	QVLQGLVFVGAHK	13	Non-ACP	0.4766	ACP	0.631
5	WRPELPPPDLPK	12	Non-ACP	0.4891	ACP	0.5159
6	HWVQMPPSGMFK	12	ACP	0.5225	Non-ACP	0.3798
7	MLPPGGYYWDR	11	ACP	0.5557	Non-ACP	0.4891
8	HFSLLMGSLFK	11	ACP	0.5077	ACP	0.6218
9	LHPDFSSSLLK	11	Non-ACP	0.3752	ACP	0.5794
10	MAMLWDPRDDR	11	Non-ACP	0.4538	ACP	0.5609
11	VVLLPLGGPAR	11	Non-ACP	0.4822	ACP	0.6966
12	DLLLNHLHPWK	11	Non-ACP	0.4363	ACP	0.5711
13	TLPDTLTEWK	10	Non-ACP	0.4323	ACP	0.5552
14	LTPDTLTEWK	10	Non-ACP	0.4259	ACP	0.6
15	SDPLLLPLLK	10	ACP	0.515	ACP	0.8213
16	LYVPQAYRWK	10	ACP	0.5612	ACP	0.6028
17	AMPLLLLPLK	10	ACP	0.5454	ACP	0.8253
18	GFQVVQPARK	10	Non-ACP	0.3925	ACP	0.5233
19	ATATNAEMYR	10	Non-ACP	0.3802	ACP	0.5855
20	WVLFGFFPGR	10	ACP	0.6034	ACP	0.7262
21	HFFPDELWK	9	ACP	0.522	ACP	0.5942
22	WLGNFPEPR	9	ACP	0.5073	Non-ACP	0.5081
23	TFHDTATPR	9	Non-ACP	0.4784	ACP	0.6241
24	DLFVLVMMR	9	ACP	0.5432	Non-ACP	0.4574
25	LLGPHRGVR	9	Non-ACP	0.4037	ACP	0.5145
26	GFVVGDHVR	9	Non-ACP	0.4493	ACP	0.6179
27	TFGPYTNAR	9	Non-ACP	0.3669	ACP	0.5372
28	YSEHAYPSK	9	Non-ACP	0.4497	ACP	0.5481
29	LVPLGSLLK	9	ACP	0.5523	ACP	0.8112
30	VFVSPGLEK	9	Non-ACP	0.4142	ACP	0.5631
31	NSLDLLHWR	9	Non-ACP	0.4464	ACP	0.5299
32	ELGPVLLLR	9	Non-ACP	0.4478	ACP	0.6011
33	LDSPLQMWK	9	ACP	0.5121	ACP	0.6523
34	VLPEVFEHK	9	Non-ACP	0.4625	ACP	0.5347
35	RFLAAVAPK	9	ACP	0.5159	Non-ACP	0.4542
36	FMWAAMYSR	9	ACP	0.6187	ACP	0.6
37	SATPYTYSK	9	Non-ACP	0.4879	ACP	0.7018
38	TYMWPPANR	9	ACP	0.5722	ACP	0.6616
39	WLGPAATPR	9	Non-ACP	0.4897	ACP	0.6688
40	FGVLLQAPK	9	Non-ACP	0.4581	ACP	0.5811
41	EVVVPLKK	8	ACP	0.5258	ACP	0.6353
42	VEVVLPQK	8	Non-ACP	0.4164	ACP	0.6362
43	FPEPVLVK	8	ACP	0.5002	ACP	0.6295
44	TDTFFNHR	8	Non-ACP	0.4429	ACP	0.5917
45	AVLGPLLK	8	ACP	0.531	ACP	0.871
46	KAEQVPWK	8	Non-ACP	0.4466	ACP	0.6226
47	WVMHLEPK	8	ACP	0.5021	Non-ACP	0.4553
48	DLALHVHK	8	Non-ACP	0.3524	ACP	0.5127
49	FFPEDLWK	8	ACP	0.5271	ACP	0.6959
50	VEALHVHK	8	Non-ACP	0.4108	ACP	0.6439
51	MFVQFTLK	8	Non-ACP	0.4481	ACP	0.5337
52	AVLGPLLK	8	ACP	0.531	ACP	0.871
53	DGWLPVPK	8	Non-ACP	0.4731	ACP	0.5618
54	NNAHVLHK	8	Non-ACP	0.3853	ACP	0.5884
55	TDTFFNHR	8	Non-ACP	0.4429	ACP	0.5917
56	KGSLLDPK	8	Non-ACP	0.3294	ACP	0.523
57	MLVVRPLR	8	ACP	0.5123	ACP	0.5661
58	MVVLEMMR	8	Non-ACP	0.4746	ACP	0.553
59	LPLLPLLK	8	ACP	0.5675	ACP	0.7105
60	VDTVLPLK	8	Non-ACP	0.4101	ACP	0.53
61	MDPPLLWR	8	ACP	0.6041	ACP	0.6961
62	TYNAKFYK	8	ACP	0.5178	ACP	0.6208
63	FGLVSVPR	8	Non-ACP	0.4853	ACP	0.6416
64	LTVGPLTK	8	Non-ACP	0.3892	ACP	0.5232
65	FFPEDLWK	8	ACP	0.5271	ACP	0.6959
66	VVMLPFFR	8	ACP	0.5845	ACP	0.5499
67	WVMHLEPK	8	ACP	0.5021	Non-ACP	0.4553
68	FFPENNWK	8	ACP	0.5797	ACP	0.6316
69	LWDLVKPR	8	ACP	0.5051	ACP	0.6331
70	QFAPLFLK	8	ACP	0.5929	ACP	0.7371
71	RANMPRAK	8	Non-ACP	0.3497	ACP	0.5257
72	MFAFDFHK	8	Non-ACP	0.4741	ACP	0.5742
73	MLSASGSK	8	Non-ACP	0.3128	ACP	0.6286
74	LWDLVQPR	8	Non-ACP	0.4407	ACP	0.5506
75	LLNLLPR	7	Non-ACP	0.4294	ACP	0.6567
76	MLLELAR	7	Non-ACP	0.406	ACP	0.5744
77	MLLELAR	7	Non-ACP	0.406	ACP	0.5744
78	LALLSQK	7	Non-ACP	0.3908	ACP	0.6906
79	LLDDLLK	7	Non-ACP	0.331	ACP	0.675
80	LLDDLLK	7	Non-ACP	0.331	ACP	0.675
81	LPPVLPR	7	ACP	0.5395	ACP	0.5947
82	HHVPVAK	7	ACP	0.5579	ACP	0.7224
83	DLVVPLK	7	ACP	0.5172	ACP	0.5848
84	VTTPPLK	7	Non-ACP	0.4781	ACP	0.5423
85	ALLPSMK	7	Non-ACP	0.4951	ACP	0.6904
86	DLVVPLK	7	ACP	0.5172	ACP	0.5848
87	EPNLLPR	7	Non-ACP	0.3636	ACP	0.5232
88	LVYSVPK	7	Non-ACP	0.4665	ACP	0.5228
89	LSADTWK	7	Non-ACP	0.4728	ACP	0.6968
90	WLSVVPR	7	ACP	0.5624	ACP	0.5675
91	LALQFVR	7	Non-ACP	0.478	ACP	0.5234
92	LALLQSK	7	Non-ACP	0.4606	ACP	0.7293
93	QPVYPWK	7	Non-ACP	0.4994	ACP	0.6283
94	MFMVTYR	7	ACP	0.5219	ACP	0.6157
95	MLEMSSK	7	Non-ACP	0.4204	ACP	0.5768
96	LVYPVSK	7	Non-ACP	0.4945	ACP	0.547
97	MLEMSSK	7	Non-ACP	0.4204	ACP	0.5768
98	LPPLVPR	7	Non-ACP	0.4929	ACP	0.5448
99	DLLPLLR	7	Non-ACP	0.496	ACP	0.6599
100	LLTHVMK	7	Non-ACP	0.4609	ACP	0.5138
101	NMMYHWK	7	ACP	0.5915	ACP	0.5983
102	ELQLALK	7	Non-ACP	0.4174	ACP	0.6298
103	LTSQFYK	7	Non-ACP	0.4452	ACP	0.5353
104	ALGYNNK	7	Non-ACP	0.3125	ACP	0.562
105	YFTWLHK	7	ACP	0.5759	ACP	0.5697
106	QQLALLR	7	Non-ACP	0.4272	ACP	0.5309
107	YDMVTYR	7	Non-ACP	0.3997	ACP	0.6572
108	QMWVPNK	7	Non-ACP	0.4776	ACP	0.5299
109	YDLVFYK	7	Non-ACP	0.4436	ACP	0.5672
110	VTEWDYK	7	Non-ACP	0.4635	ACP	0.5493
111	DLLLHTR	7	Non-ACP	0.3646	ACP	0.5372
112	LYEWSLK	7	ACP	0.5251	ACP	0.6027
113	EPPEPRR	7	Non-ACP	0.4117	ACP	0.6196
114	MWVFPER	7	ACP	0.5971	ACP	0.6294
115	MLLLHSR	7	Non-ACP	0.4436	ACP	0.5846
116	LTVSRPR	7	Non-ACP	0.3673	ACP	0.5351
117	ANAVAVR	7	Non-ACP	0.2906	ACP	0.5421
118	YDLVFYK	7	Non-ACP	0.4436	ACP	0.5672
119	LVAATLK	7	Non-ACP	0.424	ACP	0.5182
120	DLTVVVK	7	Non-ACP	0.4075	ACP	0.5722
121	LYLDLK	6	Non-ACP	0.4018	ACP	0.558
122	FFYPGK	6	ACP	0.5371	ACP	0.5578
123	WAFPLK	6	ACP	0.7036	ACP	0.7724
124	FFYPGK	6	ACP	0.5371	ACP	0.5578
125	LYLDLK	6	Non-ACP	0.4018	ACP	0.558
126	FQVLVK	6	ACP	0.5909	ACP	0.6439
127	WVDLDK	6	Non-ACP	0.4744	ACP	0.595
128	WAFPLK	6	ACP	0.7036	ACP	0.7724
129	LLPFPR	6	ACP	0.6473	ACP	0.7133
130	WLLLTR	6	ACP	0.5993	ACP	0.6604
131	LLPFPR	6	ACP	0.6473	ACP	0.7133
132	TWDMAK	6	Non-ACP	0.4816	ACP	0.5691
133	NFLMAR	6	ACP	0.505	ACP	0.5337
134	TFPLPK	6	ACP	0.5892	ACP	0.6088
135	TFPPLK	6	ACP	0.5434	ACP	0.6122
136	MEMMFK	6	ACP	0.6083	ACP	0.6372
137	LLVSHK	6	Non-ACP	0.4603	ACP	0.6162
138	LVQDLK	6	Non-ACP	0.3525	ACP	0.5831
139	TWSETK	6	Non-ACP	0.4565	ACP	0.5924
140	LFDVYK	6	ACP	0.5263	ACP	0.558
141	MWDAPR	6	ACP	0.5559	ACP	0.6512
142	AVLDLK	6	Non-ACP	0.3807	ACP	0.6063
143	MEMMFK	6	ACP	0.6083	ACP	0.6372
144	MDLFVR	6	Non-ACP	0.4711	ACP	0.5242
145	LLVSHK	6	Non-ACP	0.4603	ACP	0.6162
146	TFPPLK	6	ACP	0.5434	ACP	0.6122
147	DEVLVK	6	Non-ACP	0.3553	ACP	0.5543
148	GFWESR	6	ACP	0.5612	ACP	0.6225
149	LYPSAK	6	Non-ACP	0.4521	ACP	0.5554
150	FMVGEK	6	Non-ACP	0.367	ACP	0.5215
151	LFEYGR	6	ACP	0.566	ACP	0.5336
152	LMMDNK	6	Non-ACP	0.391	ACP	0.5885
153	WLLLEK	6	ACP	0.601	ACP	0.7139
154	VTLPLK	6	Non-ACP	0.4849	ACP	0.6107
155	DFTDNK	6	Non-ACP	0.3698	ACP	0.5505
156	LVDKLK	6	ACP	0.5029	ACP	0.6698
157	YPSTER	6	Non-ACP	0.4175	ACP	0.5755
158	QKTVYR	6	Non-ACP	0.3541	ACP	0.6018
159	VEYSRR	6	Non-ACP	0.4061	ACP	0.613
160	RPSVHK	6	Non-ACP	0.4131	ACP	0.5125
161	YQFPPR	6	ACP	0.6398	ACP	0.6553
162	YVTAEK	6	Non-ACP	0.3849	ACP	0.5742
163	LMPMFR	6	ACP	0.6311	ACP	0.7146
164	YNFDMR	6	ACP	0.5592	ACP	0.623
165	LPATNK	6	Non-ACP	0.3289	ACP	0.5342
166	DLLMFR	6	Non-ACP	0.4615	ACP	0.6025
167	NDMFFK	6	Non-ACP	0.475	ACP	0.5974
168	ELVEHK	6	Non-ACP	0.3602	ACP	0.5234
169	TFPLPK	6	ACP	0.5892	ACP	0.6088
170	LVEEHK	6	Non-ACP	0.3509	ACP	0.5273
171	LSTLR	5	Non-ACP	0.3159	ACP	0.5415
172	LLLQR	5	Non-ACP	0.4551	ACP	0.6113
173	DLLFK	5	ACP	0.5658	ACP	0.7623
174	EVLLR	5	Non-ACP	0.487	ACP	0.5909
175	FEYGR	5	ACP	0.5605	ACP	0.6147
176	FAVER	5	Non-ACP	0.3478	ACP	0.517
177	LDELK	5	Non-ACP	0.3167	ACP	0.5829
178	LPALK	5	ACP	0.5594	ACP	0.7516
179	LQDFR	5	Non-ACP	0.4549	ACP	0.5695
180	DLLFK	5	ACP	0.5658	ACP	0.7623
181	EVLLR	5	Non-ACP	0.487	ACP	0.5909
182	NNLFK	5	ACP	0.5104	ACP	0.6131
183	EEPDK	5	Non-ACP	0.3465	ACP	0.576
184	SSWKK	5	ACP	0.6361	ACP	0.6807
185	LPLLR	5	ACP	0.5655	ACP	0.6535
186	TLLSK	5	Non-ACP	0.3775	ACP	0.6076
187	LFPLK	5	ACP	0.6311	ACP	0.7728
188	AVLVR	5	Non-ACP	0.415	ACP	0.5986
189	FEYGR	5	ACP	0.5605	ACP	0.6147
190	LTLSK	5	Non-ACP	0.3753	ACP	0.5742
191	LSLTR	5	Non-ACP	0.3739	ACP	0.562
192	SPSSK	5	Non-ACP	0.4068	ACP	0.622
193	HSSEK	5	Non-ACP	0.3297	ACP	0.5681
194	SLELK	5	Non-ACP	0.3981	ACP	0.6804
195	LSDLR	5	Non-ACP	0.3283	ACP	0.5579
196	DLLLR	5	Non-ACP	0.3909	ACP	0.5978
197	MYGTK	5	Non-ACP	0.4934	ACP	0.5467
198	EVLLR	5	Non-ACP	0.487	ACP	0.5909
199	FAMPR	5	ACP	0.5646	ACP	0.6476
200	DLVAK	5	Non-ACP	0.3628	ACP	0.5158
201	LLQLR	5	Non-ACP	0.4621	ACP	0.6139
202	YAPLR	5	ACP	0.5447	ACP	0.6267
203	VTELK	5	Non-ACP	0.3432	ACP	0.5558
204	HTAYK	5	ACP	0.5391	ACP	0.6588
205	TAVPR	5	Non-ACP	0.3708	ACP	0.5267
206	SMSMR	5	Non-ACP	0.4136	ACP	0.64
207	DLVAK	5	Non-ACP	0.3628	ACP	0.5158

ACP, anticancer peptides; MLACP, Machine-Learning-Based Prediction of Anticancer Peptides; RFACP, random forest anti-cancer peptides; SVMACP, support vector machine anti-cancer peptides.

### Cancer cells treated with crocodile sera demonstrated difference in gene expression compared with the control

**Figure 6:**
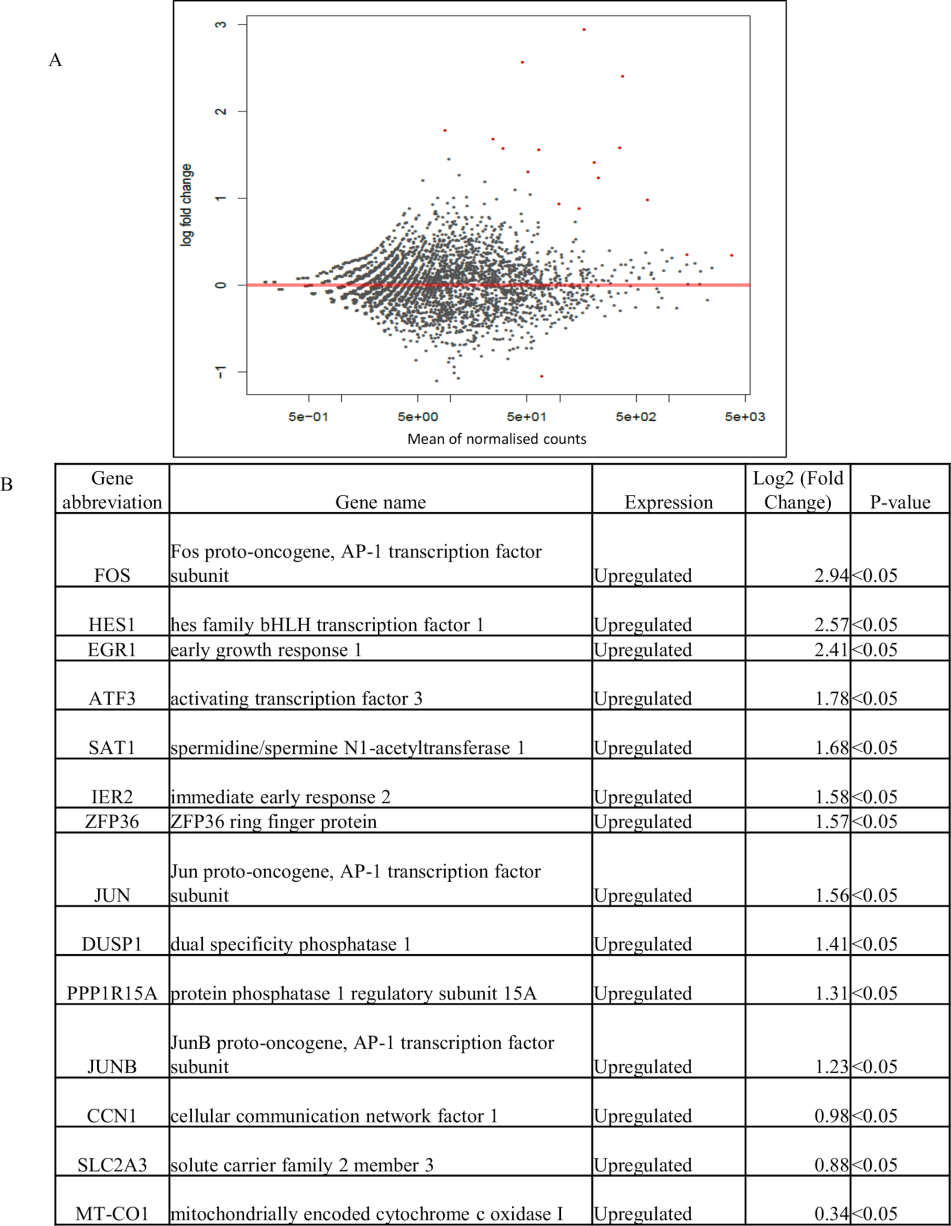
(A–B) Differential gene expression of HeLa cells treated with crocodile sera. Fourteen genes were upregulated as compared with control. The red dots in the MA plot demonstrates the expression of genes (A) (p<0.05 using Wald statistics).

**Figure 7:**
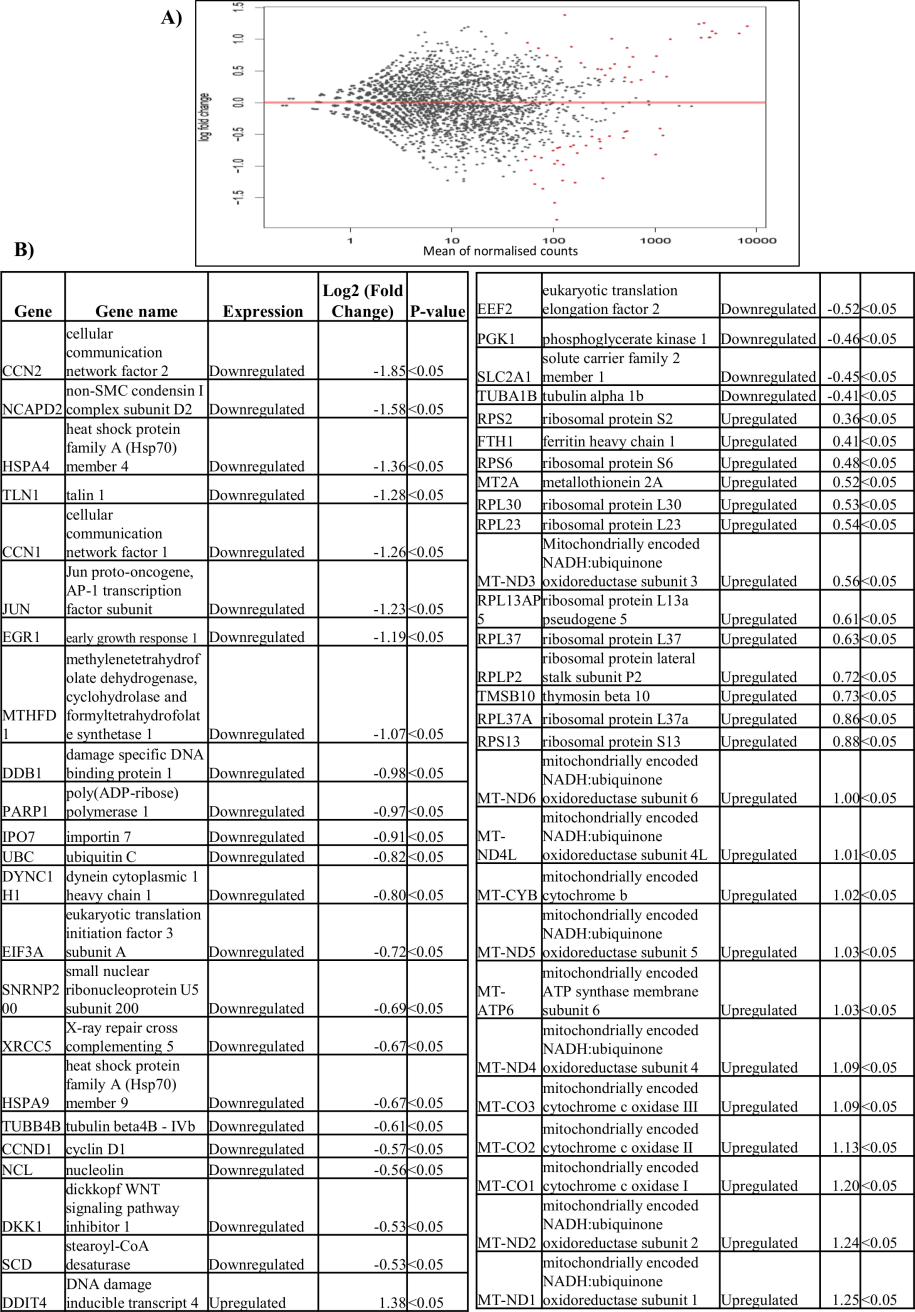
(A–B) Differential gene expression of MCF7 cells treated with crocodile sera. Twenty-six genes were downregulated and 25 genes were upregulated as compared with control. The red dots in the MA plot demonstrates the expression of genes (A) (p<0.05 using Wald statistics).

**Figure 8:**
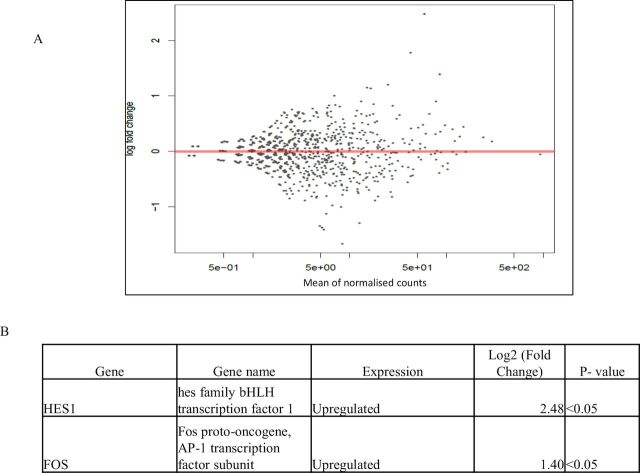
(A–B) Differential gene expression of PC3 cells treated with crocodile sera. Two genes were upregulated as compared with control. The red dots in the MA plot demonstrates the expression of genes (A) (p<0.05 using Wald statistics).

Gene expression analysis revealed the presence of 14 genes in treated HeLa cells, 51 genes in treated MCF7 cells and 2 genes in treated PC3 cells that were differentially expressed, as compared with untreated control cells (p<0.05) (figures 6–8). Treated HeLa cells demonstrated 14 genes that were upregulated as compared with the control (p<0.05) (figure 6), whereas treated MCF7 cells demonstrated 26 genes that were downregulated and 25 genes that were upregulated as compared with the control (p<0.05) (figure 7). Treated PC3 cells demonstrated two genes that were upregulated as compared with the control (p<0.05) (figure 8).

## Discussion

The mortality and morbidity of cancer remains a serious concern,^1–3^ suggesting the need for new effective anticancer agents. The medicinal properties of naturally derived products remain an important source of therapeutic drugs. Here, we dissected a saltwater crocodile (*C. porosus*) and prepared crude lysates and sera. The organ lysates and serum were then tested on cancer cells for growth inhibition and cytotoxic activity. The gall bladder lysates and serum inhibited more than 99% of HeLa cells, PC3 cells and MCF7 cells. This was consistent with previous findings that showed the ability of *C. siamensis* bile extracts in hindering the growth of human cholangiocarcinoma cells, hepatocellular carcinoma cells, ovarian carcinoma cells and gastric adenocarcinoma cells.^11 12 14^ The blood extract of *C. siamensis* and American alligator also induced cell cycle arrest that led to growth inhibition among cancer cells.^34–36^ Notably, crocodile sera but not bovine sera caused irreversible cancer cell damage.

Next, LC-MS/MS was performed on serum samples to identify the types of potential anticancer small molecules and peptides. Besides being the most convenient biological sample, serum also paves the road by being a liquid highway for all the molecules that are synthesised, secreted and discarded from the body.^29^ Using LC-MS/MS, 80 small molecules were detected and 19 compounds were putatively identified from the serum of *C. porosus* by comparison against the METLIN metabolomics database. It has been reported that the number of small molecules detected are normally lesser than 24 metabolites at a time due to the limitation of metabolite databases.^29^

Valdecoxib, a cyclooxygenase 2 (COX-2) inhibitor,^37^ which is commonly used to treat inflammation in conditions such as rheumatoid arthritis and knee osteoarthritis patients^38^ were identified in the serum of *C. porosus* (table 2). COX-2 is highly expressed in several types of cancer such as colorectal cancer.^37 38^ Besides that, chemotherapy and radiotherapy also contribute to the upregulation of COX-2 expression, resulting in the onset of resistance against cancer therapy.^39^ Therefore, the presence of valdecoxib in crocodile serum may protect the animal from cancer, therefore highlighting the fact that valdecoxib could be a potential anticancer drug, since it inhibits the expression of COX-2 and works as an anti-inflammatory agent.^38^ However, patients treated with valdecoxib previously suffered from side effects such as cardiovascular complications, leading to the withdrawal of valdecoxib from the market in 2005 by the Food and Drug Administration (FDA) agency of the United States Department of Health and Human Services and the European Medicines Agency.^37 39^ Purine has the ability in treating many conditions due to its antitumour, antiviral, anti-inflammatory, antimicrobial and antiparasitic properties.^40 41^ Previously, purine was shown to inhibit DNA replication in cancer cells^42^ although the exact anticancer mechanisms exerted by purine remain vague. FDA-approved purine antimetabolites or derivatives such as 6-mercaptopurine, fludarabine, nelarabine, cladribine, clofarabine and pentostatin have been extensively used for the treatment of cancer although the presence of selectivity and toxicity of these compounds is still questionable.^42^

LC-MS/MS analysis of the serum of *C. porosus* demonstrated seven peptides belonging to the alpha-2-macroglobulin isoform X2 (Accession ID: A0A1U7S0T0) protein family, five peptides belonging to the transferrin (Accession ID: A0A286T2Q9) protein family, six peptides belonging to the complement C3 (Accession ID: A0A1U7S0C0) protein family, three peptides belonging to the fibrinogen beta chain (Accession ID: A0A1U7SP96) protein family, two peptides belonging to the haemoglobin subunit beta (Accession ID: P86919) protein family and three peptides belonging to the serum albumin isoform X2 (Accession ID: A0A1U8CYA2) protein family (table 3). The remaining 749 detected peptides were categorised as novel peptides. The potential ACP from the list of novel peptides were then predicted using the MLACP online tool,^31^ and interestingly more than 207 ACP were predicted from serum of *C. porosus* (table 4).

It is anticipated that these ACP may be utilised in clinical treatment of cancer in the future. The mechanism of action and the anticancer activity of the 207 novel peptides detected here need to be determined and investigated further. In addition, the bioavailability and stability under physiological conditions of these peptides need to be considered. Strategies to allow appropriate delivery of peptides have been utilised in the past resulting in highly efficacious treatment. Some cancer-targeting peptides have been designed on the basis of the pH difference between tumour tissue and normal tissues,^43^ and the peptide selectively kills tumour cells at acidic pH levels but is not toxic against normal cells. Moreover, nanotechnology and nanomaterials have provided remarkable potential for application of ACP in tumour-targeted therapy, bioimaging and diagnosis due to their unique properties. The discovery of ACP and associated pharmacological research and development is noteworthy, and further investment is needed over the next several decades to exploit their potential and benefit thousands of cancer patients.

To our knowledge, this is the first study that applied differential gene analysis of cancer cells treated with crocodile serum. The gene expression analysis revealed that 14 genes in treated HeLa cells, 51 genes in treated MCF7 cells and 2 genes in treated PC3 cells were deferentially expressed as compared with untreated control cells, out of more than 10 000 genes (p<0.05) (figures 6–8). Furthermore, treated HeLa cells demonstrated 14 genes that were upregulated and no downregulated genes as compared with control (figure 6). This included Fos, a proto-oncogene, involved in important cellular events, including cell proliferation, as well as other genes such as immediate early response 2 that is a putative nuclear protein that functions as a transcription factor in cellular responses, and may be involved in the regulation of tumour progression and metastasis.^44^ Additionally, treated MCF7 cells demonstrated 26 genes that were downregulated and 25 genes that were upregulated as compared with control (figure 7). These comprised genes that are involved in cellular communication, DNA repair, growth response, respiration and so on. Treated PC3 cells demonstrated two genes that were upregulated with no downregulated genes as compared with control (figure 8). These included Fos as well as the Hes1 gene which codes for nuclear proteins that suppress transcription.^44 45^

Nonetheless, differential gene expression across the different cell lines was not consistent. The reason for this could be due to the cell lines having different properties and they are of different origin. HeLa cells are derived from cervical cancer cells.^46^ These cells proliferate abnormally rapidly, even compared with other cancer cells. PC3 cells on the other hand were established from bone metastasis of grade IV of prostate cancer. These cells do not respond to androgens, glucocorticoids or fibroblast growth factors, but results suggest that the cells are influenced by epidermal growth factors.^47^ On the other hand, MCF-7 cells are one of the very few cells known to express substantial levels of the oestrogen receptor alpha.^48^ Future studies should be conducted on several cell lines of similar origin to determine if there is a conserved pathway in response to the lysates tested in this study. These findings show that animals living in polluted environments possess molecules that have potential anticancer activities. Consequently, it is important to investigate the anticancer effects of these compounds against various cancer cells and in vivo. In summary, we showed that the organ lysates and sera of *C. porosus* exhibit potent anticancer activity and have identified several molecules that could serve as potential drug leads, but further research is needed to realise these expectations. These findings further suggest that animals residing in polluted milieus are a large unexploited source for prospective pharmaceutical drugs, and could lead to the identification of novel antitumour compound(s) and/or understanding of the mechanisms of cancer resistance.
